# Dissecting the Genetics of Forage Yield and Related Traits in Soft Red Winter Wheat

**DOI:** 10.3390/cimb48100973

**Published:** 2026-09-22

**Authors:** Maria Ali, Ali Missaoui, Madhav Subedi, Suraj Sapkota, Ali Babar, Mohamed Mergoum

**Affiliations:** 1 Department of Crop & Soil Sciences, University of Georgia, Athens, GA 30602, USA; ma85310@uga.edu (M.A.); cssamm@uga.edu (A.M.); 2 Institute of Biotechnology, Cornell University, Ithaca, NY 14853, USA; ms3838@cornell.edu; 3 Small Grains and Potato Germplasm Research Unit, Aberdeen, ID 83210, USA; suraj.sapkota@usda.gov; 4 Agronomy Department, Institute of Food and Agricultural Sciences, University of Florida, Gainesville, FL 32611, USA; mababar@ufl.edu

**Keywords:** dual-purpose wheat, forage yield, GWAS, QTL, marker-assisted selection

## Abstract

The use of wheat as forage remains underutilized, particularly in the southeastern U.S., despite its potential to fill seasonal forage gaps when warm-season forages are dormant. Genetic improvement of forage yield traits in wheat has been limited. A genome-wide association study on 182 soft red winter wheat lines was conducted to identify quantitative trait loci for five forage yield traits. The panel was evaluated across four environmental-year combinations in Georgia (2023–2025). ANOVA revealed significant phenotypic variation, with heritability ranging from low to moderate in combined locations and from low to high within individual locations. Correlation analysis revealed significant positive associations between growth stages (GS), forage height (FHt), fresh forage weight (FWt), and dry forage weight (DWt). Using 47,339 SNP markers, we identified 192 marker–trait associations. Twenty-two major-effect QTLs explained 10% of the phenotypic variance, of which six were multi-trait. Chromosomes 7A, 1B, and 6B harbor key regions for FHt, FWt, and DWt. Accumulating favorable alleles showed significant increases in FHt (7 cm), FWt (1611.2 kg/ha), DWt (58.6 kg/ha), and germination counts (G) (4.5%), while reducing GS (0.81 units). QTLs (*QFht.uga-7A*, *QFht.uga-4A*) were possibly allelic with known loci, and the remaining 20 QTLs appeared novel. Candidate genes were identified for 10 of 22 QTLs related to plant growth and development. This study provides promising candidates for forage yield improvement in wheat under forage-production systems, supporting marker-assisted selection. Upon further validation, these results can be used in breeding for desirable plant architecture to improve wheat potential for forage yield.

## 1. Introduction

Winter wheat is a cool-season annual grass that is a valuable source of energy for humans (grains) and for feed (forage or grain) in the U.S. Southern Great Plains. Despite its substantial standing biomass and high nutritional value, it is less frequently used as a pasture in the southeast (SE) U.S. states [1,2]. In the SE, perennial forage systems such as bahiagrass (*Paspalum notatum* Flueggé), bermudagrass (*Cynodon dactylon* L.), and tall fescue (*Schedonorus arundinaceus* Schreb.) are extensively utilized to fulfill the forage needs of the beef industry [3]. Consequently, the issue arises of ensuring sufficient green forage with high nutritional value for lactating and growing animals when warm-season perennial pastures grow slowly or go dormant, and biomass becomes scarce in the fall and early spring. In fact, due to cold and freezing weather conditions, warm-season forages like alfalfa (*Medicago sativa* L.) and switchgrass (*Panicum virgatum* L.) become completely dormant in the winter. Meanwhile, wheat acreage has steadily declined in Georgia (GA) due to relative commodity prices and profitability, a trend similar to that in other SE states [4]. Therefore, winter wheat could be a promising option for producers in the SE region because of its exceptional winter hardiness and extended growth period. This can provide grazable forage during the period from late fall through the following spring when all other crops are dormant [5]. In this production system, wheat can be managed solely for either forage, for grain, or as a dual-purpose crop that supports both grazing and grain production within the same growing season.

Forage harvest at different plant developmental stages plays a crucial role in achieving high-yielding forage, and the right balance in this regard enables growers to enhance profitability [6]. Forage clipping delayed until late joint (Zadoks 32) or boot (Zadoks 40) stages typically led to a substantial increase in average forage yield from 3000 kg/ha to 5000 kg/ha [7]. Given that jointing marks the onset of vigorous plant growth, delaying forage harvest at this developmental stage can substantially boost forage production [7,8], but it will subsequently reduce the quality. Plant height is an important trait in forage-based production systems as biomass accumulation is positively associated with canopy height and can therefore serve as an indirect indicator of forage yield [9,10]. However, in the case of dual-purpose wheat systems, a trade-off often exists between harvesting forage at later developmental stages and maintaining forage quality and subsequent grain yield. Delayed forage harvest can reduce nutritive value and negatively affect grain yield potential by increasing competition for assimilates and altering developmental dynamics [6,8,11].

Despite the importance of wheat as a forage resource, improvements in forage yield and nutritive value have received relatively limited emphasis in U.S. wheat breeding programs. To our knowledge, no information is available on the use of genome-wide association studies (GWAS) to identify quantitative trait loci (QTLs) governing forage yield and related traits in wheat. However, extensive work has been performed on wheat for other agronomic and grain and yield-related traits, such as plant height and biomass at grain harvest [12,13,14,15,16]. Therefore, this GWAS was conducted to investigate the genetic basis of forage yield and related traits in soft red winter wheat (SRWW). The ultimate goal is to develop high-yielding cultivars with improved nutritive value available during the period when warm-season and perennial forages go dormant and the availability of green forage becomes scarce in the SE U.S. The specific objectives of the study were to (1) evaluate SRWW elite germplasm for forage yield and related traits using a diverse GWAS panel across multiple environments in the SE region, (2) utilize the GWAS to identify QTLs for these traits, and (3) postulate candidate genes controlling major-effect QTLs that are stably expressed in diverse environments.

## 2. Material and Methods

### 2.1. Planting Material and Experimental Method

A diverse panel composed of 182 SRWW advanced genotypes was used in this study. The panel was developed by public breeding programs across multiple states in the SE U.S., thereby encompassing a broad spectrum of genotypes adapted to the diverse environmental conditions characteristic of the SE region. The experimental trials were conducted at three different locations in GA over two years (2023–2025): the University of Georgia Southwestern Research and Education Center (SWREC) in Plains during the first year (2023–2024) and the SWREC in Plains, GA (32° 2.80′ N, 84° 21.98′ W), the J. Phil Campbell Sr. (JPC) Research and Education Center in Athens, GA (33° 52.2′ N, 83° 25.8′ W), and the Iron Horse Farm (IHF) in Watkinsville, GA (33° 51.5′ N, 83° 23.5′ W) during 2024–2025. They were referred to as GAP_24, GAP_25, JPC_25, and IHF_25, respectively. A randomized complete block design with two replications was used for this study across all locations. Each genotype of the diversity panel was planted in a plot with 7 rows, each row 3 m long and 7 inches apart, at a seeding rate of ~140 kg ha^−1^. At each location, the soil was prepared before planting and fertilized with nitrogen, phosphorus, and potassium according to site-specific soil test recommendations. Planting was done across years during October and November each year, with GAP_24 (29 November), GAP_25 (22 October), IHF_25 (24 October), and JPC_25 (25 October), respectively. All plots were sown at a seed rate of 65 g per plot using a GPS-equipped tractor (John Deere, Moline, Illinois, USA; Trimble Technologies, Westminster, CO, USA) coupled with an auto-trip seed drill (Hege Equipment Inc, Salt Lake City, UT, USA).

### 2.2. Data Collection: Phenotypic Traits

The GWAS panel was phenotyped for five forage yield and related traits, including germination counts (G), Zadok’s growth stages at forage harvest (GS), plant height at forage harvest in cm (FHt), fresh/dry forage weight in kg/ha (FWt), and (DWt) for two growing seasons (2023–2025). G was determined for each genotype by visually counting emerged plants 2–3 weeks after planting. GS was assessed using the Zadoks GS scale [17] at forage harvest. FHt was measured as the average length from the plant base to the growing point in cm. Forage was clipped at 8–10 cm above the soil surface, and total biomass was quantified immediately post-harvest. FWt was measured immediately after harvesting the entire plot with a Swift forage harvester at the end of tillering and before stem elongation [18]. Plot-level FWt was converted to kg/ha based on the harvested plot area. DWt was subsequently calculated from the oven-dried forage biomass and expressed as kg/ha. The procedure was applied consistently across all locations and years. Each location and year combination was treated as an independent environment. For instance, GAP, IHF, and JPC had single environments: GAP_24, GAP_25, IHF_25, and JPC_25. In addition, a combined dataset (All-combined) was created for each trait by pooling data across all four environments. In total, there were five environments, including single and combined environments, which were considered in the analysis. The homogeneity of variance among the combined environments was tested using Levene’s test [19].

### 2.3. Data Analysis

All measured traits were analyzed using R software (v4.5.1) (RStudio, Boston, MA, USA), separately for each environment and jointly across the environments available for each trait, in a randomized complete block design with two replications per environment. Each location–year combination was treated as an environment. Each trait was analyzed separately as the response variable. By using the specific R package lme4 (v4.5.1), analysis of variance (ANOVA), mean trait values, standard deviation (SD), and coefficients of variation (CV%) were determined. Pearson correlation coefficients were calculated to assess the magnitude and direction of associations among the measured traits using the across-environment dataset, implemented with the psych package in R (v4.5.1). For single-environment analyses, the overall mean was treated as a fixed intercept, and genotype and replication were modeled as random effects (Equation (1)), and genotype, environment, and replication for the combined analysis were modeled as random effects (Equation (2))

Y*ij* = μ + G*i* + R*j* + ε*ij* (1)

where Y*ij* represents the phenotypic response observed in the *j*^th^ replication for the *i*^th^ genotype, μ is the overall mean, G*i* is the effect of the *i*^th^ genotype, R*j* is the effect of the *j*^th^ replication, and ε*ij* represents the residual error term associated with the observation Y*ij*.

Y*ijk* = μ + G*i* + (GE)*ij* + (ER)*jk* + ε*ijk* (2)

where Y*ijk* represents the phenotypic response observed for the *i*^th^ genotype in the *j*^th^ environment and *k*^th^ replication, μ is the overall mean, G*i* is the random effect of the *i*^th^ genotype, +(GE)*ij* is the random genotype × environment (G × E) interaction, (ER)*jk* is the random effect of replication nested within environment, and ε*ijk* represents the residual error term associated with the observation Y*ijk*.

Best linear unbiased predictions (BLUPs) for each trait were estimated for all single environments and the combined dataset using the (lme4) package in R [20] and were subsequently used for GWAS analysis. Broad-sense heritability (H^2^) was estimated from variance components separately for single environments (Equation (3)) and for a combined environment (Equation (4)).

(3) $$H^{2}=\frac{\sigma_{G}^{2}}{\sigma_{G}^{2}+\sigma_{e}^{2}/n}$$

(4) $$H^{2}=\frac{\sigma_{G}^{2}}{\sigma_{G}^{2}+\frac{\sigma_{GE}^{2}}{n_{E}}+\frac{\sigma_{ER}^{2}+\sigma_{e}^{2}}{n_{E}n_{R}}}$$

where $\sigma_{G}^{2}$ represents the genetic variance, $\sigma_{GE}^{2}$ represents the variation attributed to G × E interactions, $\sigma_{ER}^{2}$ refers to variation due to replications within each environment, $\sigma_{e}^{2}$ is the residual variance, and $n_{E}$ and $n_{R}$ correspond to the harmonic mean number of environments assessed per genotype and the harmonic mean number of replications within each G × E combination, respectively.

### 2.4. Quality Control and SNP Identification

The GWAS diversity panel was genotyped via genotyping-by-sequencing (GBS) at the USDA-ARS lab in North Carolina. Detailed descriptions of the genotyping procedures and quality control steps applied to this panel have been reported by Guo et al. [21]. Briefly, genomic DNA was extracted from fresh green seedling tissue and used to construct GBS libraries following digestion with the restriction enzymes *PstI* and *MseI* [22]. SNP discovery and genotype calling were performed using the Trait Analysis by aSSociation, Evolution and Linkage (TASSEL)-GBS discovery pipeline v5.2.49 [23]. SNP filtering was subsequently applied by removing markers with a minor allele frequency below 5% and those with more than 50% missing data, as described by Guo et al. [21].

### 2.5. Population Structure and Linkage Disequilibrium

Linkage disequilibrium (LD) was quantified as the squared allele frequency correlation coefficient (r^2^) between pairs of SNP markers within chromosomes, following the method of Hill and Weir [24], using the TASSEL software package (v5.0) [23]. LD estimates were calculated for marker pairs located on the same chromosome. Genome-wide and chromosome-specific LD decay patterns were visualized by plotting r^2^ values against physical distance, and a critical r^2^ threshold was used to characterize LD decay. Population structure was assessed through principal component analysis (PCA) implemented in the Genome Association and Prediction Integrated Tool (GAPIT) package in R, and the resulting principal components were included as covariates in the GWAS to account for population stratification.

### 2.6. GWAS Analysis

BLUP estimates for each trait across all four environments and the All-combined dataset (GAP_24, GAP_25, IHF_25, JPC_25, across-environment BLUP) were used in GWAS to detect significant genomic regions associated with forage yield and related traits in SRWW. GWAS analysis was performed using the GAPIT (v3.0) implemented in R [25,26]. A single model was selected by running GWAS under both single-locus and multi-locus frameworks, including the general linear model (GLM), mixed linear model (MLM), multiple-loci mixed model (MLMM), and fixed and random model circulating probability unification (FarmCPU). PCs derived from population structure analysis were included as covariates, and model performance was evaluated using quantile–quantile (Q-Q) plots and scree plots to ensure adequate control of population structure within the diversity panel.

A significance threshold of 1 × 10^−4^ [27] was applied to identify marker–trait associations (MTAs), as the Bonferroni-corrected genome-wide threshold was deemed overly stringent for the present dataset. The proportion of phenotypic variance explained (PV, adjusted R^2^) by each significant marker was estimated using an ordinary least squares regression model, with the phenotype modeled as a function of the marker genotype. Pairwise LD was assessed among significant MTAs on the same chromosome using TASSEL v5.0 [23]. Markers exhibiting LD (r^2^) values greater than or equal to the defined critical threshold were considered linked and grouped into the same putative QTL region. Putative major-effect candidate QTLs were operationally defined as those explaining ≥10% of PV and were retained for subsequent analysis and named according to the current standardized guidelines proposed by Boden et al. [28]. A QTL’s stability was determined by the presence of associated markers across at least 2 of the 5 tested environments.

#### 2.6.1. Effect of Individual Favorable Allele and Additive Allele Effect of Major-Effect QTLs

To evaluate the allelic effect of the major-effect QTLs identified in this study, the diversity panel was stratified into three genotype classes for each associated marker (favorable-allele homozygous, heterozygous, and unfavorable-allele homozygous). Favorable alleles were operationally defined as those demonstrating desirable forage yield and related phenotypes, specifically higher G, FHt, FWt, and DWt, along with lower GS. BLUPs for the associated trait were subsequently compared across genotypic classes using Tukey’s Honestly Significant Difference (HSD) test. This post hoc comparison procedure controls the experiment-wise Type I error rate at α = 0.05, ensuring robust identification of significant differences among the three genotypic groups while accounting for multiple comparisons.

For each QTL associated with a given trait, the most significant marker was selected, and genotypes were grouped based on the number of favorable alleles present, including a null class containing no favorable alleles. Wheat lines carrying these allele combinations were then identified, and their BLUP values for the corresponding trait in the across-environment BLUP were compared using Tukey’s HSD test. 

#### 2.6.2. QTLs Novelty and Candidate Gene Identification

Candidate gene identification was conducted by examining genomic regions spanning 5 kb upstream and downstream of each peak SNP using the Chinese Spring RefSeq v1.1 reference genome assembly [29] accessed through Ensembl Plants (http://plants.ensembl.org). Gene models located within or near peak markers were extracted and subjected to functional annotation. Putative gene functions were inferred using multiple complementary databases, including UniProt (https://www.uniprot.org/) for curated protein information, InterPro (https://www.ebi.ac.uk/interpro/search/sequence/, accessed on 23 March 2026) for conserved domains and functional motifs, and the Wheat Expression Browser (http://www.wheat-expression.com/) to evaluate expression profiles across tissues and developmental stages. To evaluate the novelty of the detected QTLs, the physical positions of identified loci were systematically compared with previously reported QTLs/MTAs in wheat and related cereals using the Chinese Spring reference and publicly available databases, PlantBioinfoPF (https://urgi.versailles.inra.fr/) and NCBI (https://www.ncbi.nlm.nih.gov/). This comparative approach enabled the assessment of positional overlap and facilitated differentiation between previously characterized regions and potentially novel loci.

## 3. Results

### 3.1. Phenotypic Analysis of Forage Yield and Related Traits

Significant differences were observed among the 182 SRWW for the five forage yields and related traits evaluated across environments in GA, USA (Supplementary Table S1, Table 1). In the All-combined dataset, G ranged from 15 to 100% (mean 80.79%, SD 11.22, CV 13.89%), with the highest mean G (89.60%) recorded for IHF_25, followed by GAP_25 (86.33%) and JPC_25 (66.48%). However, genotypic differences were not significant in any single environment nor in the combined analysis (Supplementary Table S1). In contrast, GS exhibited strong evidence of genetic control and pronounced environmental effects. While GS ranged from 23 to 37, with a relatively tight distribution in the combined dataset (mean 29.02, SD 1.17, CV 4.03%) (Supplementary Table S1, Table 1), the single-location mean GS ranged from 25.27 to 31.88, with the highest (31.88) mean observed for GAP_24. Single-environment ANOVA for GS detected significant genotypic effects at IHF_25, GAP_25, and GAP_24 (*p* < 0.001), while JPC_25 showed non-significant genotypic differences (Supplementary Table S1). The All-combined analysis also showed a highly significant genotype main effect and a significant G × E interaction (*p* < 0.001).

FHt also showed significant variation, ranging from 13.0 to 68.1 cm, with a combined mean of 33.3 cm (SD 4.38, CV 13.16%) (Table 1). However, the highest mean FHt was observed at GAP_24 (48.88 cm). Genotypic effects were highly significant at IHF_25, GAP_25, and GAP_24 (*p* < 0.001), whereas at JPC_25, genotypic differences were non-significant (Supplementary Table S1). The combined ANOVA identified highly significant genotype main effects (*p* < 0.001) and a G × E interaction (*p* < 0.001). FWt displayed the widest relative dispersion in the combined dataset (range 556.59 to 20,525.72 kg/ha and a mean of 6258.10 kg/ha) (Table 1), with the highest mean FWt of 8105.52 kg/ha observed at JPC_25. Genotypic differences were significant at IHF_25 (*p* < 0.05), GAP_25 (*p* < 0.001), and GA24 (*p* < 0.01), but at JPC_25 they were non-significant (Supplementary Table S1; Figure 1). In the All-combined analysis, FWt retained a significant genotype effect (*p* < 0.05) alongside a highly significant G × E interaction (*p* < 0.001). Finally, DWt ranged from 175.77 to 5312.08 kg/ha (mean 1814.61 kg/ha) (Table 1; Figure 1), with the highest DWt mean value (2724.21 kg/ha) observed at IHF_25 (Supplementary Table S1). Significant genotypic differences were detected for DWt only at GAP_24 (*p* < 0.01), with non-significant genotype effects in other environments (Supplementary Table S1). Importantly, in the All-combined analysis, both the genotype main effect (*p* = 0.9604) and G × E (*p* = 0.5508) were non-significant (Supplementary Table S1).

Broad-sense heritability (H^2^) estimates for forage yield and related traits varied widely among traits in the combined dataset (Table 1). Overall, H^2^ values ranged from 0.002 to 0.229, indicating relatively low H^2^ for forage yield and related traits across environments. Among the evaluated traits, FHt exhibited the highest H^2^ (0.229), indicating a moderate proportion of phenotypic variation explained by genetic factors. Correspondingly, G and GS showed lower H^2^ estimates (0.095 and 0.172, respectively), indicating greater environmental influence on these traits. Similarly, both FWt and DWt showed very low H^2^ of 0.182 and 0.002, respectively (Table 1). This reflects the complex genetic inheritance of these traits and the strong environmental and substantial G × E effects for these traits. H^2^ estimates at individual locations further demonstrated varying environmental influences, ranging from 0.01 to 0.64 for GS and FHt at GAP_24, GAP_25, JPC_25, and IHF_25. However, H^2^ for FWt and DWt was generally low to moderate (0–0.39) across all locations (Supplementary Table S1).

Correlation analysis was performed using across-environment BLUPs for 182 SRWW lines (Figure 2). Significant positive correlations (*p* < 0.001) were observed among all forage yield and related traits except G, which did not show significant associations with GS, FHt, FWt, and DWt. The strongest significant correlation was detected between GS and FHt (r = 0.80), followed by the relationship between FHt and FWt (r = 0.66). GS also exhibited significant positive correlations with FWt and DWt (r = 0.60 and r = 0.39, respectively). In addition, FHt was significantly positively associated with DWt (r = 0.38), and FWt showed a significant positive correlation with DWt (r = 0.50) (Figure 2). Correlation among traits at single locations also revealed the same pattern, with G showing no significant correlations across all locations (GAP_25 and JPC_25) except IHF_25, where G showed significant positive correlations with GS, FHt, and DWt (Supplementary Figure S1). Overall, a significant positive correlation was observed between GS and FHt, FWt, and DWt across all environments (GAP_24, GAP_25, IHF_25) except at the JPC_25 location (Supplementary Figure S1).

### 3.2. Population Structure and LD Analysis

A total of 47,339 high-confidence SNP markers distributed among the three subgenomes were retained for downstream analyses. The B subgenome contained the highest marker density, with 23,679 SNPs (50%), followed by the A subgenome with 17,055 SNPs (36%), whereas the D subgenome harbored only 6605 SNPs (14%). LD decay analysis conducted across the whole genome revealed an average LD half-decay distance of approximately 6.36 Mb (Supplementary Figure S2). The A genome exhibited the most rapid LD decay, with a half-decay distance of 2.80 Mb, followed by the D subgenome at 5.13 Mb, while the B subgenome showed the slowest LD decay, extending to approximately 10 Mb. Substantial variation in LD decay was also observed among individual chromosomes, with the shortest decay distance detected on chromosome 4D (41 bp) and the longest on chromosome 1D (43 Mb) (Supplementary Figure S2). An r^2^ threshold of 0.2 was used to define LD between marker pairs. Accordingly, markers located on the same chromosome with pairwise r^2^ values ≥ 0.2 were considered linked and grouped into the same putative QTL. Population structure was evaluated using principal component analysis (PCA) based on genome-wide SNP data. The scree plot of the first ten PCs showed a sharp decline in the proportion of variance explained by the first four components, followed by a gradual leveling off, indicating that these PCs captured the major population structure within the panel. Consequently, the first four PCs were included as covariates in the GWAS models to account for population stratification.

### 3.3. GWAS Results

Genome-wide association analyses were conducted using 47,339 high-confidence SNPs and five phenotypic datasets: BLUPs estimated separately for each of the four field environments (GAP_24, GAP_25, IHF_25, and JPC_25) and genotypic BLUPs estimated jointly across all four environments (across-environment BLUP). The across-environment BLUP dataset was used to examine average genetic effects across the tested environments and was not treated as a fifth independent environment. GWAS was performed on four different models (GLM, MLM, MLMM, and FarmCPU). FarmCPU model was then selected based on the distribution of SNPs in Q-Q plots and the number of significant SNPs identified by the models (Supplementary Figure S3). FarmCPU iteratively fitted associated markers as pseudo-QTNs in its fixed-effect model and evaluated the selected pseudo-QTNs through its random-effect component, thereby accounting for relatedness within the FarmCPU framework [25]. All other FarmCPU parameters were maintained at their default GAPIT version 3 settings. FarmCPU was fitted separately to each phenotypic dataset and did not include marker × environment interaction terms; these analyses were not designed to identify loci underlying G × E interaction directly. Differences in MTA detection among the analyses provide descriptive evidence of environmental dependence but do not constitute a formal statistical test of marker × environment interaction.

A total of 192 MTAs were detected across all five datasets (significance threshold of *p* < 1 × 10^−4^) for the evaluated forage yield and related traits (Supplementary Table S2; Table 2). Among the datasets, GAP_25 had the highest number of MTAs (97), followed by across-environment BLUP (35), GAP_24 (33), and JPC_25 (20). In contrast, IHF_25 attributed the fewest MTAs (7), all of which were associated exclusively with FWt. Across the wheat genomes, the A genome harbored the largest proportion of MTAs (109), followed by the B genome (77), while the D genome had the fewest MTAs (6) (Table 2; Supplementary Table S2).

Among the 192 MTAs identified, 103 were associated with GS, representing the trait with the highest number of MTAs across four datasets (Table 2; Supplementary Table S2). In addition, 6 other MTAs were shared with FHt. GAP_25 accounted for the majority of GS MTAs (83), whereas no MTAs were detected for GS in IHF_25. These GS MTAs were distributed across eight chromosomes, with chromosome 1A containing the highest number (78 MTAs), and the single largest effect for GS, explaining up to 19.3% of PV, was attributed to marker S7B_887145 on chromosome 7B. FWt was associated with 47 MTAs across all five datasets, distributed across six chromosomes, including 1 MTA shared with DWt (Supplementary Table S2). JPC_25 and GAP_24 exhibited the largest number of FWt MTAs, each 13, explaining up to 12.8% PV, followed by across-environment BLUP with 12 MTAs explaining up to 6.7% PV. The marker S1B_533829236, located in JPC_25 on chromosome 1B, explained the highest PV of 12.8% for FWt. Furthermore, 10 MTAs were identified for DWt across three datasets (GAP_24, JPC_25, and across-environment BLUP). These MTAs were distributed across seven chromosomes, with each dataset containing markers explaining more than 10% PV (Table 2, Supplementary Table S2). No DWt MTA was detected in more than one of the four independent single-environment analyses. Because the across-environment BLUP was calculated from the same four field trials, detection in this analysis was not considered independent replication. Accordingly, the DWt MTAs are reported as dataset-specific candidate associations rather than reproducible associations across environments. A total of 15 MTAs were detected for FHt from four datasets excluding IHF_25, whereas across-environment BLUP had 6 MTAs that were associated with MTAs found for GS (Supplementary Table S2). Among these, GAP_24 and across-environment BLUP contained markers explaining more than 10% PV (14.5% and 11.7%, respectively). In addition, G identified 17 MTAs across two datasets (GAP_25 and across-environment BLUP), with each dataset containing at least one marker explaining more than 10% of PV (Table 2; Supplementary Table S2).

The MTAs for all forage yield and related traits were identified across 16 chromosomes, but none were detected on chromosomes 3D, 4D, 5D, 6D, and 7D (Table 2; Supplementary Table S2). Chromosome 1A had the highest number of MTAs (78), explaining up to 12.8% PV, followed by chromosome 1B (28 MTAs, up to 13.4% PV). Subsequently, chromosomes 7A and 6B each had 14 MTAs, accounting for 14.5% and 10.2%, respectively, and chromosome 2B (11) had 11 MTAs, accounting for 13.0%. Additionally, chromosomes 7B, 3B, 5A, 5B, and 2D each had fewer than 10 MTAs, with maximum PV values of 19.3%, 13.4%, 7.2%, 4.8%, and 3.1%, respectively (Supplementary Table S2; Table 2).

### 3.4. PutativeMajor-Effect QLTs for Forage Yield and Related Traits

The 192 significant MTAs were resolved into 68 putative QTLs based on the specific LD critical value ≥ 0.2 (Supplementary Table S2). Furthermore, we identified 22 major-effect candidate QTLs with PV ≥ 10% (Table 3). The QTL on chromosome 7A, *QFht.uga-7A*, had a single marker and was associated with FHt (Table 3) and was identified in one environment (GAP_24) and across-environment BLUP, explaining 10.0–10.9% PV. Another two major-effect QTLs (*QFht/Gs.uga-7A.2* and *QFht/Gs.uga-7A.3*) on chromosome 7A were detected for FHt and GS, showing co-localization within the same genomic region at GAP_24 and across-environment BLUP. *QFht/Gs.uga-7A.2* was associated with one different marker (S7A_10270159) and was associated with FHt in GAP_24 and across-environment BLUP, explaining 11.7% and 12.0% of PV, and GS in across-environment BLUP, explaining 8.6% PV (Table 3). Whereas *QFht/Gs.uga-7A.3* had two markers, in which S7A_10867659 was found for FHt (detected in GAP_24), explaining 14.5% PV while S7A_10909138 was associated with GS (detected in across-environment BLUP), explaining 9.4% PV (Table 3). There was another major-effect QTL for FHt (*QFht.uga-4A*) on chromosome 4A, which was detected in a single environment (GAP_24). This QTL was linked to a single marker (S4A_717822349), explaining 12.7% of PV. Interestingly, all three major-effect QTLs on chromosome 7A were identified for FHt; however, two major-effect QTLs were associated with multiple traits (for FHt and GS) and were co-located in the same genomic region. Overall, four QTLs related to FHt on chromosomes 4A and 7A explained PV of up to 14.5% (Table 3).

Chromosome 2B harbored another major-effect QTL, *QGs.uga-2B*, which was associated with GS (Table 3). This QTL for GS was identified in one environment (GAP_24) and across-environment BLUP. The QTL explained 12.8% of the PV for GS in GAP_24 and 10.1% in across-environment BLUP (Table 3; Supplementary Table S2). Two single-marker major-effect QTLs, *QGs.uga-1A.4* (S1A_16789687) and *QGs.uga-7B* (S7B_887145) on chromosomes 1A and 7B, respectively, were associated with GS and identified in the GAP_25 environment, explaining 12.8% and 19.3% of PV, respectively. Notably, the QTL on 7B had the largest PV effect on forage yield and related traits. Another two major-effect QTLs (*QGs.uga-1B* and *QGs.uga-6A*) located on chromosomes 1B and 6A for GS were identified at GAP_25 and JPC_25, explaining up to 11.1% and 11.0% PV, respectively (Table 3).

A total of 7 major-effect QTLs were identified and associated with FWt. Three major-effect QTLs, *QFwt/Dwt.uga-1B.1*, *QFwt/Dwt.uga-1B.2*, and *QFwt/Dwt.uga-1B.3* on chromosome 1B were associated with both FWt and DWt and showed co-localization within the same genomic region (Table 3; Supplementary Table S2). *QFwt/Dwt.uga-1B.1* and *QFwt/Dwt.uga-1B.2* were related to multiple markers, explaining respectively up to 10.6% and 12.0% PV for FWt while up to 9.6% and 9.3% PV for DWt. However, *QFwt/Dwt.uga-1B.3* was linked to one marker (S1B_534667041) controlling both FWt and DWt at JPC_25, explaining 11.4% and 10.4% of PV, respectively. Similarly, chromosome 6B *QFwt/Dwt.uga-6B.2* major-effect QTL controlling both FWt and DWt explained up to 10.2% PV for FWt and 9.2% PV for DWt (Table 3; Supplementary Table S2). Chromosome 6B also harbored another major-effect QTL (*QFwt.uga-6B.2*) for FWt, explaining up to 10.2% of the PV. Two other major-effect QTLs (*QFwt.uga-1B.2* and *QFwt.uga-1B.3*) on chromosome 1B were found to be associated with FWt, explaining 12.1% and 12.8%, respectively, of PV (Table 3). Thus, all 7 major-effect QTLs for FWt were located on chromosomes 1B and 6B. Chromosome 1B harbored the highest number of major-effect QTLs (8), of which 5 were associated with FWt, and all five were identified in the JPC_25 environment; the remaining 2 major-effect QTLs for FWt were observed in GAP_24 (Table 3; Supplementary Table S2). On the other hand, we also identified two major-effect QTLs for DWt on chromosome 1D and 3B (*QDwt.uga-1D* and *QDwt.uga-3B*) in the GAP_24 environment and across-environment BLUP, each harboring a single marker and explaining 12.0% and 10.6% PV, respectively (Table 3). Although these DWt-associated loci met the operational major-effect criterion of PV ≥10%, none was reproduced in at least two independent single-environment analyses. Thus, the major-effect designation refers only to the PV threshold and does not indicate cross-environment validation. Considering the extremely low across-environment H^2^ of DWt, these loci should be treated as promising candidates for marker-assisted selection pending validation.

Four major-effect QTLs were identified for G, two of which were located on chromosome 1B (*QG.uga-1B.5* and *QG.uga-1B.6*) in the across-environment BLUP, explaining 13.4% and 10.6% PV, respectively. The other two QTLs were identified on chromosomes 2A and 3B (*QG.uga-2A* and *QG.uga-3B.2*) from the GAP_25 environment and explained up to 14.5% and 13.4% of PV, respectively (Table 3). Overall, we identified 22 major-effect QTLs for all studied forage yield and related traits (Table 3). Among them, six were multi-trait QTLs, co-located in the same genomic regions. In addition, we identified another small-effect but stable QTL (≥2 environments) on chromosome 7A, *QGs/Fht.uga-7A.1* (with LD critical value ≥0.2 threshold) explaining up to 8.2% PV. Small-effect QTLs *QGs/Dwt.uga-2D* and *QDwt/Fwt.uga-3B* were also found to be associated with multiple traits, including GS, FWt, and DWt, and explained up to 7.4% PV (Supplementary Table S2).

### 3.5. Effect of Individual Alleles

Markers associated with eight of the major-effect QTLs, *QGs.uga-1A.4*, *QFwt.uga-1B.2*, *QFwt/Dwt.uga-1B.3*, *QFwt.uga-1B.3*, *QDwt.uga-1D*, *QG.uga-2A*, *QFht.uga-4A*, and *QGs.uga-7B*, were examined for their allelic effects (Table 4). Genotype-class sizes were highly unbalanced for several markers, particularly for some heterozygous and low-frequency homozygous classes. Therefore, genotype classes represented by fewer than 10 wheat lines were retained in Table 4 to describe the observed genotype distribution and mean BLUP values but were treated as descriptive only and were not used to support robust biological inference regarding allelic effects. For markers with adequately represented homozygous genotype classes, differences in BLUP values between favorable and unfavorable homozygotes supported the direction of the allelic effects identified in the GWAS (Table 4). Consequently, interpretation focused primarily on adequately represented homozygous classes rather than sparsely represented heterozygous classes. The lines with the homozygous state of the favorable allele “C” (CC, 19 lines) for the QTL *QFwt.uga-1B.2* (S1B_526750430) had 302.71 kg/ha higher FWt mean values than the lines with the homozygous unfavorable allele “T” (TT, 162 lines). Similarly, for QTL *QFwt.uga-1B.3* associated with FWt (S1B_533829236), the difference between the homozygous state of the favorable allele (TT, 17 lines) and the unfavorable allele (CC, 153 lines) was 322.24 kg/ha (Table 4). A multi-trait QTL (*QFwt/Dwt.uga-1B.3*) with a single marker (S1B_534667041) had a favorable allele “T” for both FWt and DWt, where the homozygous favorable allele (TT, 19 lines) had 341.77 kg/ha higher FWt and 48.82 kg/ha higher DWt means in comparison to the homozygous unfavorable allele (CC, 151 lines). In addition to that, QTL *QDwt.uga-1D* (S1D_354847019) for DWt, where the lines carrying the homozygous favorable allele (CC, 63 lines) had a mean DWt value 78.12 kg/ha higher than that of lines carrying the homozygous unfavorable allele (TT, 112 lines) (Table 4).

The QTL *QGs.uga-7B* (S7B_887145) exhibited the largest allelic effect for GS, where the homozygous state of its favorable allele (CC, 163 lines) contributed to the decrement of GS by 1.15 mean value in comparison to the homozygous unfavorable allele (AA, 3 lines) (Table 4). Similarly, *QGs.uga-1A.4* (S1A_16789687) had the favorable allele “T” (TT, 148 lines), contributing to a 0.52-unit decrement in GS compared to the homozygous unfavorable allele (CC, 9 lines) (Table 4).

QTL for G (*QG.uga-2A*) was associated with marker S2A_43254369 (Table 4). The lines homozygous for the favorable alleles (TT, 88 lines) exhibited an increase in mean G by 0.91% compared to the lines with homozygous unfavorable alleles (GG, 78 lines) (Table 4). For FHt, *QFht.uga-4A* showed the largest allelic effects across three markers. The marker S4A_717822349 was associated with the favorable allele “A,” where homozygous lines for the favorable allele (AA, 37 lines) had a mean FHt 3.64 cm higher than those with the homozygous unfavorable allele (GG, 132 lines) (Table 4). However, the heterozygous class (GA) contained only three lines and was not used to infer a heterozygous effect.

### 3.6. Favorable-Allele Accumulation of Major-Effect QTLs

The genotypes included in the association panel originated from multiple breeding programs and were not developed through targeted strategies to combine specific loci. As a result, the allele configurations detected at the significant QTL represent naturally occurring combinations within the germplasm. The favorable-allele analysis was therefore conducted as an allelic-effect evaluation within the GWAS discovery population to characterize phenotypic differences associated with the accumulation of favorable alleles. Because the QTLs and the direction of their favorable effects were identified using the same phenotypic dataset, this analysis does not represent independent validation or establish causal effects of allele accumulation, and the magnitude of the observed differences may be subject to selection-related inflation. The analysis therefore characterizes associations between naturally occurring favorable-allele combinations and trait expression within the present panel rather than deliberate QTL pyramiding through controlled crosses. We evaluated favorable-allele accumulation at major-effect QTLs associated with DWt, FHt, FWt, G, and GS to characterize potentially useful allelic patterns for forage or dual-purpose wheat breeding. Based on the number of favorable alleles accumulated, we identified four groups each for DWt and G, five groups each for FHt and FWt, and six groups for GS (Figure 3). For all traits examined, wheat lines carrying the highest number of favorable alleles exhibited significantly (*p* < 0.05) greater trait values compared with lines lacking favorable alleles.

For GS, there were no wheat lines lacking favorable alleles (null). Lines carrying a single favorable allele exhibited higher GS, whereas stacking two to six favorable alleles resulted in a significant reduction in GS (Figure 3). Within the discovery population, accumulation of favorable alleles at QTLs *QGs.uga-1A.4*, *QGs.uga-1B*, *QGs.uga-2B*, *QGs.uga-6A*, and *QGs.uga-7B* was associated with an observed reduction of 0.81 GS units across the corresponding favorable-allele classes. Similarly, no wheat lines were identified with no favorable alleles for G. Lines carrying four favorable alleles exhibited higher G relative to those with only one favorable allele. The combined effect of favorable alleles from QTLs *QG.uga-1B.5*, *QG.uga-1B.6*, *QG.uga-2A*, and *QG.uga-3B.2* resulted in a 4.5% increase in G (Figure 3).

For FHt, the highest values were observed in lines with all four favorable alleles stacked, whereas reduced FHt was observed in the null allele group. Favorable alleles from QTLs *QFht.uga-4A*, *QFht/Gs.uga-7A.2*, *QFht.uga-7A*, and *QFht/Gs.uga-7A.3*, collectively increased FHt by approximately 7 cm (Figure 3). Similarly, no wheat line was identified with either 1 or 3 favorable alleles for FWt. However, by stacking seven favorable alleles for major QTLs (*QFwt/Dwt.uga-1B.1*, *QFwt/Dwt.uga-1B.2*, *QFwt/Dwt.uga-1B.3*, *QFwt.uga-1B.2*, *QFwt.uga-1B.3*, *QFwt.uga-6B.2*, and *QFwt/Dwt.uga-6B.2*) significantly increased FWt by 1604.60 kg/ha (Figure 3). Furthermore, seven wheat lines carrying three stacked favorable alleles exhibited significantly higher DWt values than the null group. The combined favorable alleles from QTLs *QFwt/Dwt.uga-1B.1*, *QFwt/Dwt.uga-1B.3*, *QDwt.uga-1D*, and *QDwt.uga-3B* resulted in an increase of 59.81 kg/ha in DWt on average (Figure 3).

### 3.7. Candidate Gene Identification

Positional candidate genes were identified within the specified ±5 kb search windows around peak SNPs representing 10 of the 22 major-effect QTLs. These QTLs are *QFwt/Dwt.uga-1B.2*, *QGs.uga-1B*, *QFwt/Dwt.uga-1B.3*, *QG.uga-1B.5*, *QG.uga-1B.6*, *QG.uga-2A*, *QGs.uga-2B*, *QFwt.uga-6B.2*, *QFht/Gs.uga-7A.3*, and *QGs.uga-7B* (Table 3). QTLs cluster on chromosome 1B (*QFwt/Dwt.uga-1B.2*, *QGs.uga-1B*, *QFwt/Dwt.uga-1B.3*) associated with FWt, DWt, and GS was co-localized with genes encoding regulatory and catalytic proteins. These regions were associated with genes annotated as Oligopeptide transporters, zinc-ribbon/protein kinase/Thioredoxin domain-containing proteins, and histone-related proteins (Table 3). Other loci on chromosome 1B (*QG.uga-1B.5*, *QG.uga-1B.6*) associated with G were linked to a Caffeoyl-CoA O-methyltransferase and oxidase N-terminal domain-containing protein. However, proteins associated with candidate genes for two FWt and DWt MTAs (S1B_532065919, S1B_532396302) have not yet been characterized (Table 3). The QTL *QG.uga-2A* for G was associated with candidate genes *TraesCS2A02G090400* and *TraesCS2A02G090500*, which encode protein kinase domain-containing proteins and Suppressor of cAMP receptor (SCAR)/WASP family verprolin-homologous complex-associated proteins (WAVE). The QTL on chromosome 2B for GS (*QGs.uga-2B*) was associated with candidate genes (*TraesCS2B02G446000*, *TraesCS2B02G446100*, *TraesCS2B02G446200*) encoding an RNA recognition motif (RRM)-containing protein; however, the proteins associated with the latter two candidate genes were not characterized yet (Table 3). FWt-associated QTL on 6B (*QFwt.uga-6B.2*) harbored *TraesCS6B02G323400*, which encodes a B-box-type zinc finger and CCT domain-containing protein (Table 3). A multi-trait QTL on chromosome 7A (*QFht/Gs.uga-7A.3*), which was associated with FHt and GS, the MTA linked to GS harbored candidate genes (*TraesCS7A02G027200* and *TraesCS7A02G027300*), which encode a Pentacotripeptide-repeat-containing protein of the PRORP family and a USP domain-containing proteins, respectively. Lastly, proteins associated with the candidate gene (*TraesCS7B02G002300*) for GS on chromosome 7B (*QGs.uga-7B*) have not yet been characterized (Table 3).

## 4. Discussion

### 4.1. Phenotypic Analysis of Forage Yield and Related Traits

We evaluated five forage yield and related traits (G, GS, FHt, FWt, and DWt) in a GWAS study with 182 SRWW lines conducted across two years and four environments and the All-combined dataset. The analysis revealed significant phenotypic variation (Table 1, Supplementary Table S1), providing a strong foundation for selecting and improving forage yield in SRWW. G showed considerable variation and diversity across environments, though no significant genotypic differences were detected in either single-environment or combined analyses, with very low H^2^. This suggests that G is predominantly influenced by environmental factors such as seedbed quality, soil temperature, and moisture, rather than by genetic differences. This finding is consistent with the work of Lollato et al. [30], who noted that wheat varieties exhibit varying abilities to germinate under hot soil conditions. This is true for our study at the GAP experiment station, located further south in GA, where different climatic conditions, including higher temperatures, prevailed during the growing seasons (https://www.wunderground.com/). Our results partly corroborate this, as the mean G at IHF_25 was higher than at GAP_24 and GAP_25, aligning with the environmental factors influencing seasonal growing conditions. In the case of GS, significant genotypic variation was observed across environments, indicating that GS is primarily influenced by genotype, although environmental factors also play a crucial role, as reflected by the significant G × E interaction in the combined analysis. The higher GS observed at GAP_24 showed that this environment was favorable for the growth of many lines. The variation in GS across environments shows that, while genetic differences between genotypes govern GS, factors such as temperature and moisture significantly influence it. These findings align with Hoffman et al. [31], who underscored the significant effect of environmental factors on GS. The lack of significant genotypic differences observed at JPC_25 suggests that this environment limited the expression of maturity differences, most likely due to overall suboptimal growth conditions. Given that GS affects forage harvest timing, the strong genotype effect and significant G × E interaction emphasize the need to tailor phenological selection to specific production systems/environments as reported by Wang et al. [32].

Significant phenotypic variability and genotypic and G × E interactions were observed for FHt, consistent with previous research reporting plant height as a highly variable trait with moderate to high H^2^ contributing significantly to biomass accumulation [33]. The highest mean FHt was observed at GAP_24, where we also observed higher GS, which can be a major factor of higher FHt in this environment. Similar results were reported by Bo et al. [34] and Jungers et al. [10], who described the relationships between GS and FHt and their subsequent effects on forage FWt and DWt. Interestingly, we observed the largest relative inconsistent environmental variation (CV > 34%) for FWt. This probably highlights the combined effects of stand establishment, tillering, plant height, and tiller density, all of which vary substantially among genotypes and environments in winter wheat [30]. At JPC_25, we observed the highest mean FWt (8105.52 kg/ha) despite no significant genotypic differences being observed, suggesting that favorable environmental factors at this location, including soil fertility, management and moisture, likely enhanced biomass production for all genotypes. Our results were consistent with the findings reported by Islam et al. [35], Kim and Anderson [36], and Atis and Akbar [37], who observed significantly higher forage production across different environments due to different soil types and significantly lower soil nutrients. However, under high-yielding environments, limited differences among genotypes have been observed for grain yield in multi-environmental trials [38]. DWt was moderately variable overall but, unlike FWt, did not show significant genotype or G × E effects in the combined dataset; it did express a significant difference in only one environment (GAP_24). This decoupling of FWt and DWt responses is consistent with studies that showed that dry matter yield in winter cereals is strongly influenced by GS and drying conditions and may thus be more environmentally driven than fresh biomass [34,35,36]. The high DWt mean at IHF_25, despite non-significant genotypic effects, suggests that conditions at this site favored higher dry matter concentration across genotypes, possibly due to delayed forage harvest timing caused by climatic conditions. Similar environment-driven differences in dry matter yield have been reported in cereal–legume forage systems, where harvest at later stages or under drier conditions increases DWt with limited change in genotypic ranking [36,39]. Given the apparent environmental dominance for DWt in this population, selection for dry biomass may be more effective if based on correlated traits with high genetic control, such as FHt or FWt, or if trials are conducted under standardized moisture conditions [40].

Overall, our results confirm that forage yield in SRWW is a complex polygenic trait influenced by both genotype and environment, with significant G × E interactions. In addition, traits such as GS and FHt showed consistent genetic effects across environments, which are promising for selection using genomic prediction models. In contrast, traits mainly affected by environmental conditions, such as G and DWt, may demonstrate their potential through appropriate agronomic management.

### 4.2. Correlation Between Forage Yield and Related Traits

Correlations among across-environment genotype BLUPs showed a highly significant GS-FHt association (r = 0.80) and significantly positive FHt-FWt and GS-FWt associations (r = 0.66 and 0.60, respectively). However, G showed no significant association with the other traits in the across-environment analysis. Relationships varied among environments: at JPC_25, the GS-FHt correlation was weak and non-significant (r = 0.20), whereas FWt and DWt were highly correlated (r = 0.97). Our results were consistent with coordinated development and biomass accumulation. Wheat and triticale studies have shown that flowering-time loci can influence biomass and its partitioning among vegetative and reproductive structures [41,42,43]. A higher Zadoks score denotes more advanced development at assessment; it does not itself demonstrate delayed heading or maturity. The consistent positive correlations between these traits highlight the expected biological coupling of height and biomass accumulation. Taller plants often exhibit higher leaf area index and stem mass, which contribute to both FWt and DWt, a pattern also observed in a study by Jing et al. [44]. Accordingly, the FHt/GS co-associations on chromosome 7A in the present study are consistent with developmental or architectural effects and do not, by themselves, demonstrate direct biomass effects. The FWt/DWt co-associations on chromosomes 1B and 6B involve measured biomass traits, but their independence from phenology remains unresolved. Moreover, the strong positive association between FHt and FWt aligns with the biomass partitioning concept: greater structural growth typically translates into higher fresh biomass yields [45]. Despite these general trends, variation in correlation magnitudes suggests genotype-specific growth dynamics. For example, traits in some germplasm sets exhibited more moderate associations, possibly reflecting differences in growth habit, resource allocation patterns, or environmental interactions. Similar trait variability has been reported in oat germplasm studies, in which correlations between plant height and yield components varied among genotypes depending on adaptation and phenological stages [46].

For dual-purpose breeding, higher forage biomass must be considered alongside nutritive value and subsequent grain production. Heading-to-dough harvests of winter-wheat hay have shown increased dry biomass together with lower crude protein and more undigested fiber [47]; separate cutting-time experiments demonstrate that later forage removal can reduce subsequent grain yield [11]. Grain responses nevertheless depend on defoliation management and growing conditions [48]. Subsequent grain yield after forage harvest was not evaluated in the present study, and these analyses do not establish the effects of the biomass-associated alleles on forage nutritive value. Forage nutritive value and subsequent grain yield after forage harvest were evaluated separately and are reported in a companion manuscript submitted for publication [49], Ali et al., submitted].

### 4.3. Novelty of PutativeMajor-Effect QTLs

The primary objective of this study is to quantify the contribution of key forage yield-related traits in wheat and to provide a basis for mapping genomic regions associated with these traits, including forage yield. The QTLs identified in this study provide promising candidate genomic regions for further investigation; however, their utility for marker-assisted selection must be established through validation in independent populations and additional environments. Based on the operational criterion of PV ≥10%, we identified 22 putative major-effect QTLs for forage yield and related traits across the four single-environment and across-environment BLUP analyses. To evaluate whether these QTLs corresponded to previously reported genomic regions, we compared their LD-supported physical intervals, rather than lead-marker positions alone, with published QTLs and MTAs after harmonizing positions to a common IWGSC Chinese Spring reference assembly. A locus was considered previously reported when its physical/LD-supported interval overlapped a published QTL/MTA interval for the same or a closely related trait. Loci for which no such overlap was detected under these operational criteria are described here as “putatively novel” rather than definitively novel. This distinction is important because variation among studies in marker density, LD structure, population background, trait definition, and reference assembly can cause biologically related loci to be represented by different physical coordinates. Accordingly, putative novelty in the present study refers to the absence of an identified overlapping locus under our stated comparison criteria and does not establish the presence of a previously unknown causal gene. Among our most noteworthy QTLs, a cluster of major-effect QTLs for FHt and GS on chromosome 7A mapped at 19.0 Mb (*QFht.uga-7A*), 10.3 Mb (*QFht/Gs.uga-7A.2*), and 10.9 Mb (*QFht/Gs.uga-7A.3*). The latter two QTLs were co-located and associated with both FHt and GS, suggesting that this genomic region may contribute to the genetic relationship between plant height and developmental stage. Together, these loci explained significant PV for FHt (up to 14.5% individually) and 9.4% PV for GS in the across-environment BLUP analysis. This highlights that chromosome 7A is a key genomic region for controlling FHt and GS for forage production. Similar regions on chromosome 7A have been reported to play a significant role in plant height in recent grain-focused QTL studies by Zhang et al. [15] and Liu et al. [16]. However, the study was not specifically evaluated under forage management or for fresh biomass traits. The authors identified a stable QTL for plant height on 7A across multiple environments. Similarly, the study by Liu et al. [16] reported that loci affecting PH often co-localize with phenology genes and canopy architecture QTL in wheat, reflecting shared hormonal and developmental pathways. But our current study extends this evidence to a forage-oriented context, where these loci directly influence harvestable biomass at an early GS. In addition, previous studies have shown that reduced height QTL have been mapped on nearly all wheat chromosomes, including 7A, indicating that the 7A region can play an important role in the genetic control of plant height [15]. The QTL mapped at 10.3 Mb (*QFht/Gs.uga-7A.2*) and 10.9 Mb (*QFht/Gs.uga-7A.3*) did not show direct overlap with previously reported FHt/GS loci under our interval- and trait-based comparison criteria and are therefore considered putatively novel candidate regions for FHt and GS. Because these two peak positions are separated by only 0.6 Mb, their designation as independent QTLs was based on their LD relationship under the QTL-grouping criterion used in this study; markers meeting the stated LD threshold should instead be interpreted as representing a shared QTL region. In contrast, *QFht.uga-7A*, located at 19.0 Mb, co-localized with the previously reported MQTL.F2.7A.2 for thousand kernel weight interval spanning 17.4–36.9 Mb [50]. The chromosome 4A locus *QFht.uga-4A*, with a lead-marker position at 717 Mb, mapped in close proximity to the previously reported 700–717 Mb region associated with Sr22 for stem rust [51]. We therefore treat these regions as previously implicated genomic regions rather than as novel loci; however, physical co-localization does not by itself establish that the same causal variant controls the traits reported in the different studies.

A GS QTL on chromosome 2B (*QGs.uga-2B*), together with QTLs on 1A, 1B, 6A, and 7B, further highlights the polygenic but tractable nature of forage development in wheat. *QGs.uga-2B* accounted for up to 13% of the PV of GS. Previous wheat studies have mapped developmental-stage associations and evaluated flowering-time loci [43,52], providing a basis for comparison with the forage-stage GS regions identified here. The 7B major-effect QTL on GS mapped at 0.88 Mb (*QGs.uga-7B*), which accounted for 19.3% of the PV, is also noteworthy. Major-effect loci on 7B have been reported for heading date and thousand kernel weight [53], but not for forage-related traits. This region may therefore harbor forage-relevant allelic variation affecting developmental timing; however, its novelty cannot be established from the lead-marker position alone. Under our interval- and trait-based comparison, *QGs.uga-7B* is considered a putatively novel candidate for GS only where its LD-supported interval does not overlap previously reported loci for the same or closely related developmental phenotypes. The presence of previously reported heading-date or yield loci on chromosome 7B also means that pleiotropy or linkage with known developmental regions cannot be excluded without higher-resolution mapping. Collectively, these GS QTLs provide candidate genomic regions for investigating the synchronization of plant development with optimal forage harvest windows, extending previous work that has focused primarily on heading, maturity, and grain production rather than biomass harvested at the forage stage.

A total of seven putative major-effect QTLs for FWt on 1B and 6B were identified in our study. Four of these (*QFwt/Dwt.uga-1B.1*, *QFwt/Dwt.uga-1B.2*, *QFwt/Dwt.uga-1B.3*, mapped at 529–534 Mb and *QFwt/Dwt.uga-6B.2* mapped at 574 Mb) were associated with both FWt and DWt, with individual PV explaining up to 12.0% for FWt and 10.0% for DWt, respectively. However, there were two major-effect QTLs (*QDwt.uga-1D* mapped at 354 Mb and *QDwt.uga-3B* mapped at 666 Mb) on chromosomes 1D and 3B, respectively, that explained up to 12.0% PV. Our results were aligned with Soriano et al. [12] and Qaseem et al. [14], who reported genomic regions on chromosomes 1B, 3B, and 6B for above-ground biomass but not for forage fresh biomass. Based on our results, we also observed a strong correlation between FWt and DWt, indicating complex and biologically relevant QTL architecture, where loci co-located in the same genomic regions contribute to the control of both traits. This emphasizes the genetic interrelationship between forage FWt and DWt in wheat. Our study therefore provides a detailed characterization of candidate loci associated with biomass measured at the forage harvest stage and supports shared genetic control of FWt and DWt at several co-located regions. After comparing the LD-supported intervals of these loci with previously published QTLs and MTAs, we did not identify directly overlapping loci specifically reported for forage-stage FWt or DWt for several of these regions. We therefore describe loci meeting this criterion as putatively novel candidate QTLs for forage biomass rather than as completely novel genomic regions. This distinction is particularly important because broader QTLs for yield and developmental traits have previously been mapped to some of the same chromosomal regions. For example, *QFwt/Dwt.uga-6B.2*, with a peak at approximately 574 Mb, falls within the broad 506–617 Mb MQTL6B.1 interval previously reported for grain weight, heading date, maturity, and spike-related traits [54]. This physical overlap does not establish that the same causal locus controls forage biomass and these grain-related traits, but it precludes an unqualified claim of absolute genomic novelty and raises the possibility of pleiotropy, linkage, or multiple functional variants within the broader region. We concluded from our results that chromosome 1B alone harbored eight major-effect QTLs, including five for FWt. This confirms that our study on yield and related traits repeatedly highlighted that the 1B chromosome harbors major QTLs for forage yield and related traits, including plant height, demonstrating its importance for early wheat biomass accumulation in forage production [13]. The low across-environment DWt H^2^ does not make the detection of a marker association mathematically impossible because H^2^ summarizes the aggregate genotypic contribution across environments, whereas the separate GWAS analyses test the effect of each marker within a particular phenotypic dataset. An allele could therefore exhibit an environment-dependent association that contributes little to repeatable variation across all environments. However, statistical significance within one dataset does not demonstrate a robust or transferable genetic effect. None of the DWt loci were detected in at least two independent single-environment analyses, and the across-environment BLUP analysis does not provide independent replication because it was derived from the same field trials. Accordingly, the DWt loci identified in this study should be considered provisional, dataset-specific candidates. Their effects must be evaluated in independent populations and additional multi-environment trials before their usefulness for marker-assisted selection can be determined. Future studies should also employ multi-environment GWAS models that explicitly test marker main effects and marker × environment interactions to distinguish persistent marker effects from environment-dependent or crossover effects.

Lastly, we identified four major-effect QTLs for G, despite limited genotypic variance at the phenotypic level, two on 1B (*QG.uga-1B.5* mapped at 29.4 Mb and *QG.uga-1B.6* mapped at 9.2 Mb), one on 2A (*QG.uga-2A* mapped at 43.2 Mb), and one on 3B (*QG.uga-3B.2* mapped at 508.9 Mb), each explaining 10.6–14.5% PV. Given the weak evidence for repeatable genotypic variation in G, unstable or false-positive associations cannot be excluded, and these regions should be regarded as provisional candidates requiring confirmation in independent populations and replicated trials. Our results were consistent with Francki et al. [55] and Czyczyło-Mysza et al. [56], who reported a significant role of the B genome in seedling germination. No previously reported QTL/MTA specifically for G or a closely related germination/establishment phenotype was identified as directly overlapping these loci under our interval-based comparison criteria; therefore, loci meeting this criterion are described as putatively novel for G. This designation is trait-specific and should not be interpreted as evidence that the corresponding genomic regions have never been implicated in other wheat traits. For example, *QG.uga-2A* at 43.2 Mb lies within the broad 21.3–63.3 Mb MQTL2A.3 interval reported for grain yield and related traits, with a reported MQTL peak region of 41.3–43.3 Mb [54]. Although the phenotypes differ and biological equivalence cannot be inferred from physical overlap alone, this comparison illustrates the need to distinguish putative trait-specific novelty from absolute genomic novelty. An additional novel aspect of this study is the explicit identification of multi-trait QTLs co-located in the same genomic regions under a forage management system. Under the stated LD-supported, reference-harmonized, and trait-based comparison criteria, 20 of the 22 major-effect QTLs were classified as putatively novel for the focal forage-associated traits. Consequently, the novelty classifications should be considered provisional until independent validation, higher-resolution mapping, and, where possible, functional analyses distinguish new causal variation from allelic variation, pleiotropy, or linkage involving previously reported loci. These co-associated regions provide candidates for further genetic characterization of forage traits in SRWW.

### 4.4. Additive Effect of Favorable Alleles from Major-Effect QTLs

The GWAS panel used in this study was not developed through deliberate QTL pyramiding; instead, favorable-allele accumulation across major-effect loci identified in the present analysis was quantified as an allelic-effect validation within the discovery population to characterize phenotypic patterns associated with naturally occurring combinations of favorable alleles. Because the same phenotypic dataset was used both to identify the significant QTLs and to define and evaluate favorable alleles, this analysis does not constitute independent validation and should not be interpreted as evidence of causal QTL effects. Moreover, selection of loci based on their associations in the discovery dataset can inflate the apparent magnitude of their effects. Therefore, the favorable-allele results presented here should be considered within-panel estimates that require confirmation in independent germplasm and environments. Our results indicate that variation in forage yield and related traits was associated with favorable alleles at multiple genomic regions distributed across the wheat genome. The allele combinations identified here may therefore provide candidate targets for subsequent validation and, if independently confirmed, potential application in genomic selection and/or marker-assisted selection [57] for wheat forage breeders. Compared with conventional phenotype-based selection, genomic selection and marker-assisted selection offer greater efficiency and precision by enabling selection based on genes/molecular markers closely linked to target genes controlling traits of interest, thereby bypassing phenotyping, reducing the influence of environmental variation, and accelerating breeding progress.

QTL pyramiding has been widely applied in wheat breeding to improve economically important traits, particularly disease resistance [58,59,60], as well as agronomic and yield-related traits [61,62]. However, studies specifically examining the pyramiding of QTLs associated with forage yield and related traits in wheat remain limited. In the context of the present study, wheat lines with increased FHt were preferred, provided that development did not exceed Zadoks GS 30, as plant height is positively associated with biomass accumulation [61,63]. Consistent with these findings, we also observed FHt positively associated with FWt and DWt. Previous work by Zhang et al. [61] demonstrated that pyramiding favorable QTLs for plant height increased height by up to 15.1 cm, thereby enhancing biomass production. Such studies support the potential value of combining favorable alleles in breeding; however, in the present study, the observed favorable-allele accumulation represents naturally occurring combinations within the GWAS discovery population rather than experimentally constructed QTL pyramids. Therefore, our results should be regarded as hypothesis-generating evidence for candidate allele combinations requiring independent validation before their breeding effects can be confirmed.

In this study, wheat lines accumulating favorable alleles from major-effect QTLs associated with G, GS, FHt, FWt, and DWt were identified. Within the discovery population, lines carrying favorable alleles at *QFht.uga-4A*, *QFht/Gs.uga-7A.2*, *QFht.uga-7A*, and *QFht/Gs.uga-7A.3* exhibited approximately 7 cm (13%) greater FHt than lines in the null favorable-allele class. Similarly, accumulation of favorable alleles at *QG.uga-1B.5*, *QG.uga-1B.6*, *QG.uga-2A*, and *QG.uga-3B.2* was associated with approximately 4.5% higher G within this panel.

For FWt, lines carrying the favorable alleles at QFwt/Dwt.uga-1B.1, QFwt/Dwt.uga-1B.2, QFwt/Dwt.uga-1B.3, QFwt.uga-1B.2, QFwt.uga-1B.3, QFwt.uga-6B.2, and QFwt/Dwt.uga-6B.2 exhibited approximately 1611.20 kg/ha (19%) higher FWt than the corresponding comparison group within the discovery population. Similarly, accumulation of favorable alleles at QFwt/Dwt.uga-1B.1, QFwt/Dwt.uga-1B.3, QDwt.uga-1D, and QDwt.uga-3B was associated with an observed difference of approximately 4% (58.59 kg/ha) in DWt.

In contrast, increasing favorable-allele accumulation at *QGs.uga-1A.4*, *QGs.uga-1B*, *QGs.uga-2B*, *QGs.uga-6A*, and *QGs.uga-7B* was associated with an observed reduction of approximately 0.81 GS units within the discovery population. Notably, we identified 30 wheat lines combining reduced GS values with FHt values of approximately 51 cm, a trait combination associated with increased forage yield and considered favorable for forage production. Overall, the accumulation of favorable alleles at major-effect QTLs was significantly associated with variation in G, GS, FHt, FWt, and DWt within the GWAS discovery population. These results identify potentially useful allele combinations for further breeding evaluation but should not be interpreted as independent confirmation of additive or causal QTL effects. Validation in independent populations and additional environments will be necessary to determine whether the observed allelic effects are reproducible and sufficiently stable for application in marker-assisted selection or QTL pyramiding.

### 4.5. Candidate Gene Discovery

In the present study, positional candidate genes were identified for 10 of the 22 major-effect QTLs associated with G, GS, FWt, and DWt. The positional candidate genes identified in this study encode proteins involved in photoperiod regulation, stress response, redox balance, hormone biosynthesis, cell wall formation, and nutrient transport. The functional annotations of these genes provide biological context for the associated QTLs; however, positional co-localization and predicted gene function alone do not demonstrate that these genes control the corresponding traits in the present panel. Therefore, the genes discussed below should be regarded as putative positional candidates rather than functionally validated causal genes. For instance, candidate gene *TraesCS6B02G323400* for FWt *QFwt.uga-6B.2* encodes a CONSTANS-like (COL) protein belonging to the CCT domain family and is characterized by one or two B-box zinc-finger domains. Members of the CONSTANS/CCT family have been implicated in photoperiodic regulation, flowering-related processes, and environmental responses in plants [64]. Thus, the annotation of *TraesCS6B02G323400* provides a plausible connection with developmental regulation, but its direct contribution to FWt or forage yield was not tested in the present study. Research in wheat supports broad associations of CCT/COL-family genes with photoperiod and flowering-related regulation, but family-level functional information does not itself establish the function of every individual member [65]. The multi-trait QTL *QFwt/Dwt.uga-1B.3* was associated with *TraesCS1B02G311300*, annotated as a probable zinc-ribbon domain-containing protein (https://www.uniprot.org/). A recent study has reported a thioredoxin H5-like annotation or drought-related context for this candidate [66]. Thioredoxin-related proteins participate broadly in cellular redox and stress-response processes; however, this information should be considered supportive functional context rather than evidence that TraesCS1B02G311300 directly determines FWt, DWt, or biomass accumulation. Functional validation of this specific locus would be required to establish such a relationship. The candidate gene *TraesCS1B02G049800* is associated with G (*QG.uga-1B.5*), which encodes caffeoyl-CoA O-methyltransferase (CCoAOMT), a key enzyme in lignin biosynthesis. CCoAOMT catalyzes methylation steps in the monolignol pathway, influencing lignin deposition and cell wall structure. Lignin strengthens cell walls and contributes to defense against diseases including *Fusarium graminearum*, the causal agent of Fusarium head blight [67]. Similarly, *TraesCS1B02G019800*, associated with G (*QG.uga-1B.6*), encodes a protein containing an NADH oxidase N-terminal domain. This gene has been grouped with adjacent loci encoding oxophytodienoate reductase-like proteins implicated in jasmonic acid biosynthesis. This pathway is central to root development and stress adaptation [68], suggesting a possible influence on germination-related traits. The candidate gene (*TraesCS2A02G090500*) identified for G-related QTL *QG.uga-2A* encodes SCAR and WAVE proteins. Members of this protein family regulate cytoskeleton dynamics through Rac/Rop GTPase signaling and are involved in polarized cell growth, including root hair and epidermal cell development [69]. For GS, the locus *QGs.uga-1B* was associated with *TraesCS1B02G401300*, annotated as an oligopeptide transporter. Such transporters facilitate nitrogen remobilization, micronutrient uptake, and detoxification processes and have been reported to be involved in tissue- and developmental-stage-specific expression patterns during wheat grain development [70]. Another GS-associated gene, *TraesCS7B02G002300*, linked to *QGs.uga-7B*, remains functionally uncharacterized in public protein databases (https://www.uniprot.org/). However, recent work by Atsbeha et al. [71] classified this locus as a resistance gene analog (RGA3) candidate in the context of yellow rust resistance.

Overall, the candidate genes identified here were prioritized based on their positions within QTL intervals and their predicted or previously reported biological functions. These annotations provide hypotheses regarding biological processes that could underlie the detected associations but do not establish causal relationships between individual genes and G, GS, FWt, or DWt. Functional validation through approaches such as gene-expression analysis in contrasting allelic classes, fine mapping, allele-specific studies, gene editing, complementation, or transgenic analysis will be necessary to determine whether these genes contribute directly to the associated phenotypes.

## 5. Conclusions

Developing cultivars for forage production while maintaining adequate yield should be a central objective of winter forage breeding programs. This study represents one of the first comprehensive genome-wide investigations of forage yield and related traits in SRWW evaluated under a forage harvest management system in the SE U.S. Substantial phenotypic variation and significant G × E interactions were observed across environments, underscoring the complex genetic architecture of forage biomass production and the strong environmental effect on studied traits. In all environments, GS, FHt, and FWt demonstrated low-to-high H^2^, suggesting these traits are amenable to genetic improvement; conversely, G and DWt showed low H^2^, emphasizing the importance of combining phenotypic selection with molecular approaches. A correlation analysis revealed a highly significant positive association between GS and fresh forage yield and related traits (FHt, FWt, DWt), highlighting the importance of plant height and biomass accumulation for forage production. This GWAS provided important insights into the genetic basis of forage yield and related traits. We identified 192 significant MTAs, which were resolved into 22 putative major-effect candidate QTLs. Among these, six were associated with multiple traits and co-located in the same genomic regions. Notably, 20 of these QTLs appear to be putatively novel, expanding current understanding of the genetic control of forage yield and related traits in wheat. Accumulation of favorable alleles at these major QTLs was associated with significant improvement of the studied forage yield and related traits in our breeding material. Putative candidate genes were discovered for 10 of the 22 QTLs, including those associated with FWt and G (e.g., *TraesCS6B02G323400* and *TraesCS2A02G090500*) that are annotated for roles in plant growth, development, and root hair/epidermal cell formation. The discovery of candidate genes enhances the biological relevance of the identified major-effect QTLs and underscores the involvement of critical regulatory and metabolic pathways in the control of forage yield and related traits. These findings provide a set of candidate genomic regions associated with forage yield and related traits under the tested management conditions. However, we emphasize that no QTL was consistently detected across all environments, so independent validation remains essential before breeding deployment. Specifically, further research using independent populations, years, locations, and forage management systems is needed to evaluate the stability and robustness of these loci. The resulting candidate regions provide a basis for further genetic and agronomic evaluation of forage performance in SRWW adapted to the southeastern U.S.

## Figures and Tables

**Figure 1:**
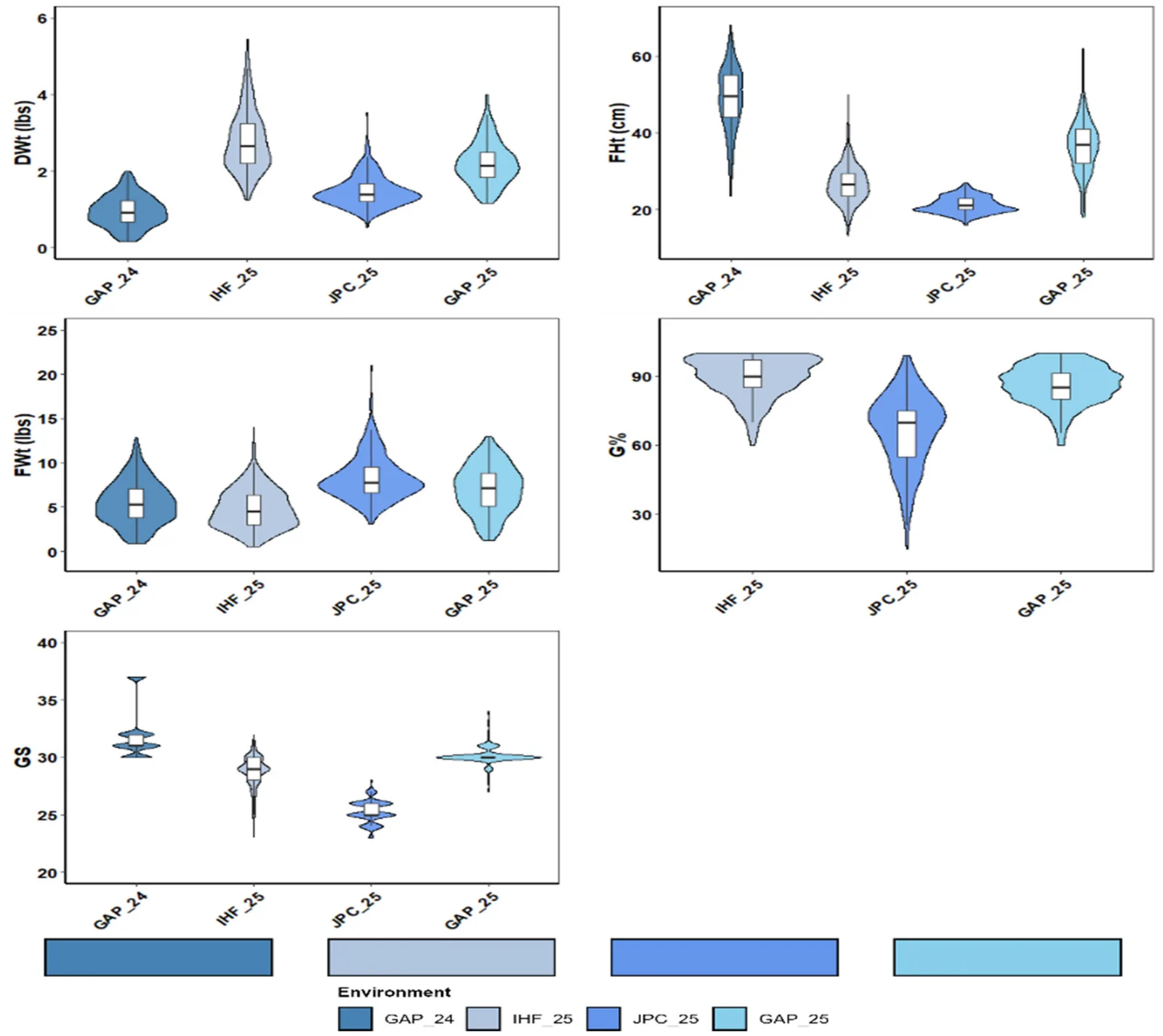
Phenotypic values of five forage yield and related traits collected in 4 single environments. Different blue-shaded violins represent different environments: GAP_24, IHF_25, JPC_25, and GAP_25, respectively. G, germination counts; GS, Zadok’s growth; FHt, forage height at harvest in cm; FWt, forage fresh weight in kg/ha; DWt, forage Dry weight in kg/ha.

**Figure 2:**
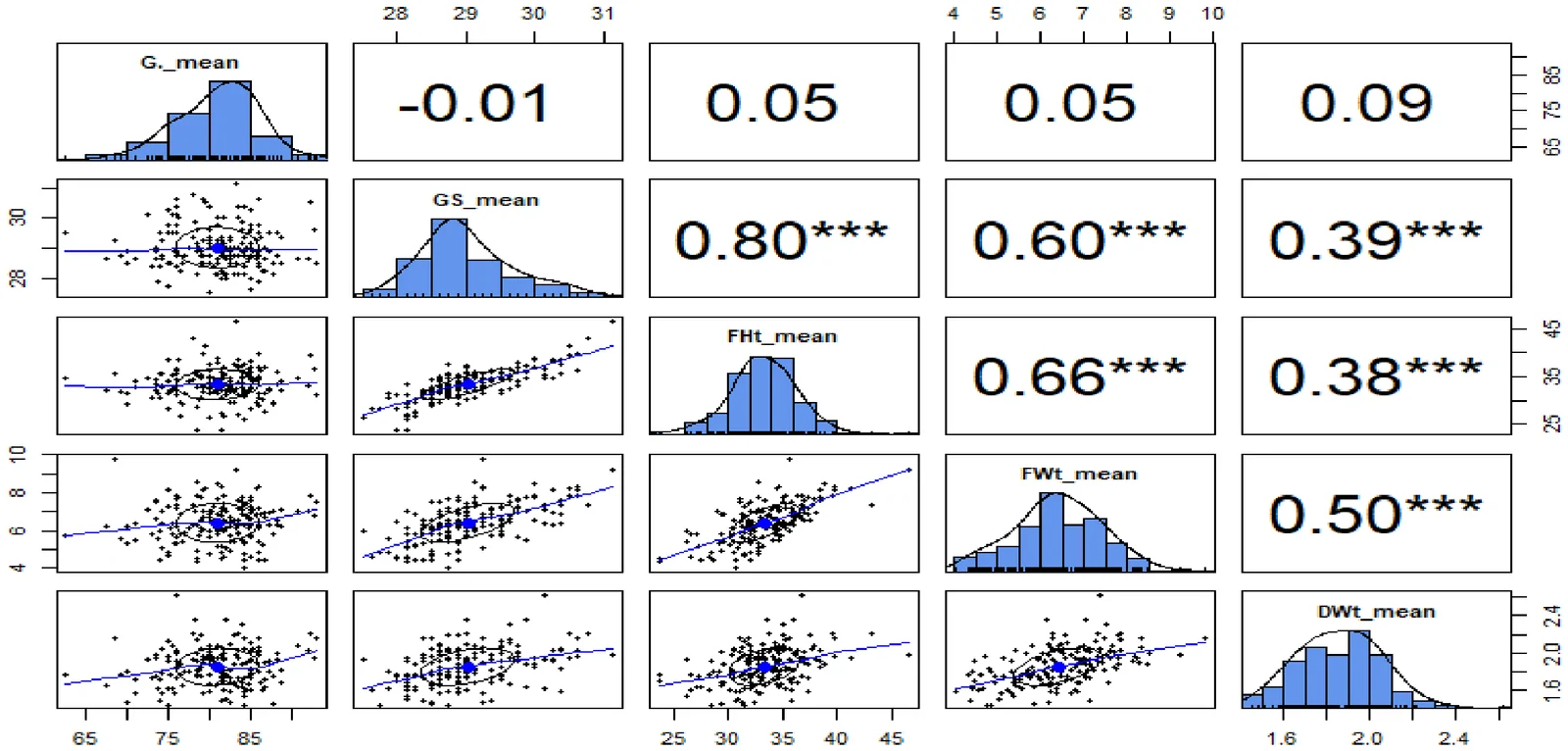
Correlation analysis of 5 forage yield and related traits among 182 SRWW lines. Combined data from three and four environments across two years, 2023–2025, and the across-environment BLUPs were used for correlation. G, germination counts; GS, Zadok’s growth stage; FHt, forage height at harvest in cm; FWt, forage fresh weight in kg/ha; DWt, forage dry weight in kg/ha; *** significant at *p* < 0.001 probability level.

**Figure 3:**
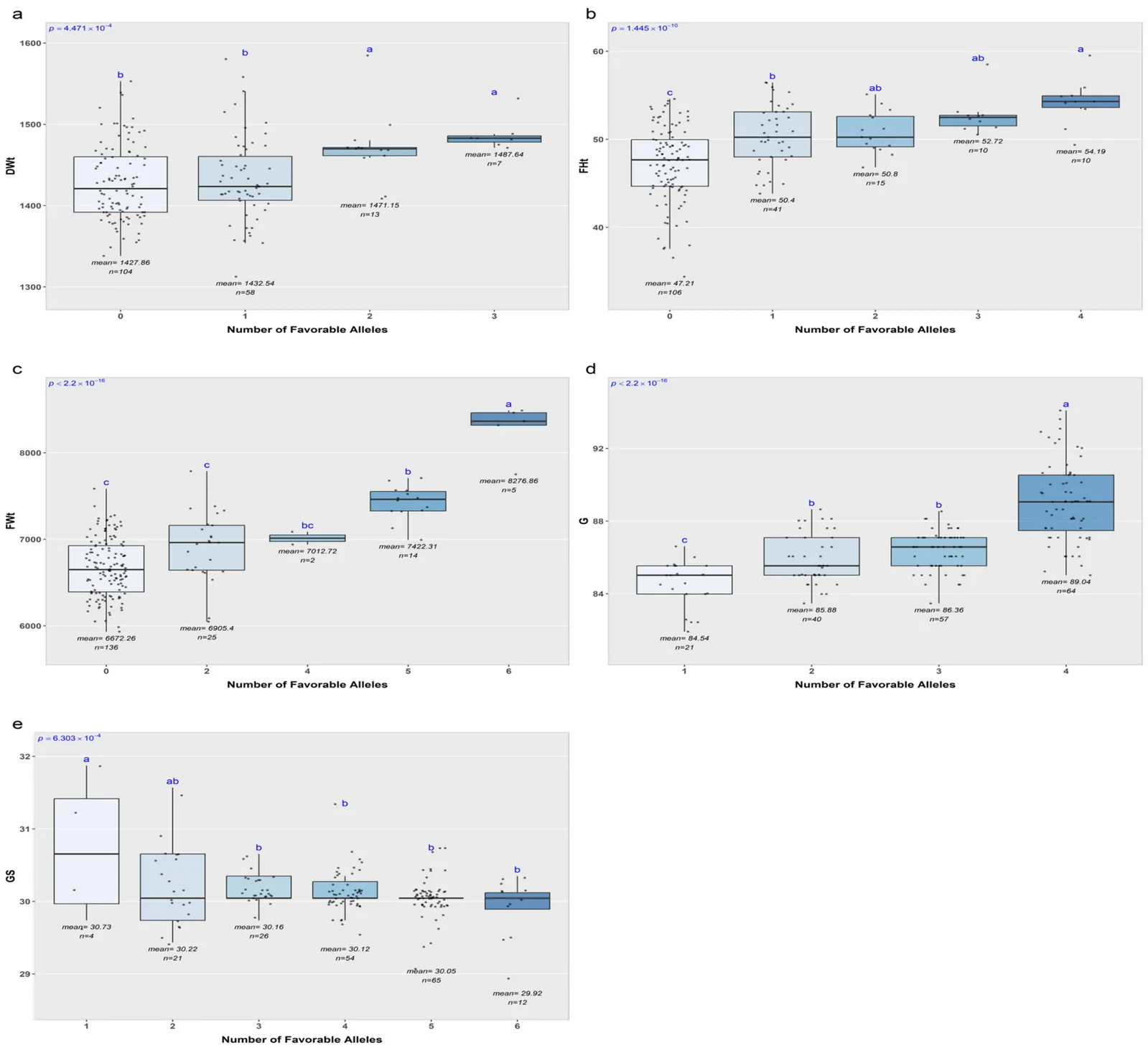
Boxplots with overlaid jittered points showing the relationship between the number of favorable alleles and the BLUP values of (**a**) dry forage weight in kg/ha (DWt), (**b**) forage height in cm (FHt), (**c**) fresh forage weight in kg/ha (FWt), (**d**) germination counts (G), and (**e**) Zadok’s growth stage (GS). Boxes represent the distributions of BLUP values for wheat lines with the indicated numbers of favorable alleles. Inddividual points represent BLUP values of individual wheat lines. Mean values are shown below each box, and the associated letter above the box shows the Tukey’s HSD results, where different letters indicate significant difference (*p* < 0.05). The value ‘N’ below each box indicates the number of wheat lines within each favorable allele class.

**Table 1:** Descriptive statistics, analysis of variance, and heritability estimation for 5 forage yield and related traits studied in 182 SRWW.

Trait ^a^	Mean	Min ^d^	Max ^e^	SD ^b^	CV ^c^	SE ^f^	H^2 g^
	----------------------------------- kg/ha-----------------------------------						
G	80.79	15	100	11.22	13.89	0.34	0.095
GS	29.02	23	37	1.17	4.03	0.03	0.172
FHt	33.30	13	68.1	4.38	13.16	0.12	0.229
FWt	6258.10	556.59	20,525.72	2129.82	34.03	55.82	0.182
DWt	1814.61	175.77	5312.08	529.86	29.20	13.89	0.002

^a^ Forage yield and related traits. G, germination counts; GS, Zadok’s growth stage; FHt, forage height at harvest in cm; FWt, forage fresh weight in kg/ha; DWt, forage dry weight in kg/ha. ^b^ SD, standard deviation from the All-combined dataset. ^c^ CV, coefficient of variation from the All-combined dataset. ^d^ Minimum value obtained for traits from the All-combined dataset. ^e^ Maximum value obtained for traits from the All-combined dataset. ^f^ SE, standard error of mean. ^g^ Broad-sense heritability estimates for the traits from the All-combined dataset.

**Table 2:** Summary of the number of MTAs for five forage yield and related traits across five environments (four single and one combined) from two years (2023–2025) studies, and chromosomes harboring MTAs across the wheat genome.

Trait ^a^	Datasets ^b^	Total MTAs ^c^	Maximum PV ^d^	Chromosomes ^e^
G	GAP_25	6	14.5	1B, 2A, 3B
	Across-environment BLUP	11	13.4	
GS	GAP_24	11	12.8	1A, 1B, 2B, 2D, 4A, 6A, 7A, 7B
	GAP_25	83	19.3	
	JPC_25	4	11.0	
	Across-environment BLUP	5	10.1	
FHt	GAP_24	7	14.5	4A, 4B, 7A, 7B
	GAP_25	6	7.9	
	JPC_25	2	9.1	
	Across-environment BLUP	0	11.7	
FWt	GAP_24	13	10.2	1B, 2B, 3B, 5A, 5B, 6B
	GAP_25	2	2.7	
	IHF_25	7	7.2	
	JPC_25	13	12.8	
	Across-environment BLUP	12	6.7	
DWt	GAP_24	2	12.0	1B, 1D, 2B, 2D, 3A, 3B, 6B
	JPC_25	1	10.4	
	Across-environment BLUP	7	10.6	

^a^ Forage yield and related traits. G, germination counts; GS, Zadok’s growth stage; FHt, forage height at harvest in cm; FWt, forage fresh weight in kg/ha; DWt, forage dry weight in kg/ha. ^b^ Datasets in which marker–trait associations were identified for each trait. Four single environments, two Plains and one each from JPC and IHF locations over two years (2023–2025), and one combined environment (a combination of all four single locations). ^c^ Number of marker–trait associations (MTAs) identified for each trait in each environment. ^d^ Maximum phenotypic variance (PV) explained among the marker–trait associations identified for each trait at each environment. ^e^ Chromosome distribution of marker–trait associations identified for each trait.

**Table 3:** Summary of 22 major-effect QTLs for five forage yield and related traits across four environments (two GAP, IHF, JPC) and across-environment BLUP over two years (2023–2025).

QTL ID ^a^	SNP ^b^	Trait_Datasets ^c^	Allele ^d^	MAF ^e^	*p* Value ^f^	Effect ^g^	PV ^h^	Candidate Gene ^i^	Protein Name ^j^ Gene Name
*QGs.uga-1A.4*	S1A_16789687	GS_ GAP_25	C/T	0.122	5.21764 × 10^−6^	−0.24	**12.82**	**-**	
*QFwt/Dwt.uga-1B.1*	S1B_529627861	FWt_ JPC_25	T/C	0.119	1.22631 × 10^−5^	−0.56	**10.56**	**-**	
		DWt_ JPC_25	C/T	0.119	3.286 × 10^−5^	−0.45	9.60	-	
*QFwt/Dwt.uga-1B.2*	S1B_531867384	FWt_ JPC_25	A/G	0.116	4.2252 × 10^−5^	−0.55	**12.01**	-	
	S1B_532065919	FWt_ JPC_25	T/G	0.116	4.2252 × 10^−5^	−0.45	**12.01**	TraesCS1B02G309100	Uncharacterized protein LOC123094139
	S1B_532396302	DWt_ JPC_25	C/T	0.116	6.5413 × 10^−5^	−0.44	9.27	TraesCS1B02G309700 TraesCS1B02G309800	Uncharacterized protein non-specific serine/threonine protein kinase, protein phosphorylation LOC123136985
*QGs.uga-1B*	S1B_631816667	GS_GAP_25	C/G	0.497	6.75464 × 10^−5^	−0.13	**11.06**	TraesCS1B02G401300	Oligopeptide transporter
*QFwt.uga-1B.2*	S1B_526750430	FWt_ JPC_25	C/T	0.113	7.90363 × 10^−5^	−0.45	**12.06**	-	
*QFwt/Dwt.uga-1B.3*	S1B_534667041	FWt _ JPC_25	T/C	0.116	1.69826 × 10^−5^	−0.67	**11.43**	TraesCS1B02G311100 TraesCS1B02G311200 TraesCS1B02G311300	Probable zinc-ribbon domain-containing protein LOC123137053 Protein kinase domain-containing protein LOC123137047 Thioredoxin domain-containing protein
		DWt_ JPC_25	C/T	0.116	4.5107 × 10^−5^	−0.78	**10.37**	TraesCS1B02G311400	E2 ubiquitin-conjugating enzyme, EC:2.3.2.23 LOC123182373
*QFwt.uga-1B.3*	S1B_533829236	FWt_ JPC_25	T/C	0.105	4.32498 × 10^−5^	−0.82	**12.82**	-	
*QG.uga-1B.5*	S1B_29492494	G_Across-environment BLUP	G/T	0.096	1.47 × 10^−5^	0.62	**13.43**	TraesCS1B02G049800	Caffeoyl-CoA O-methyltransferase
*QG.uga-1B.6*	S1B_9270181	G_Across-environment BLUP	C/T	0.253	6.8426 × 10^−5^	0.34	**10.64**	TraesCS1B02G019800	NADH:flavin oxidoreductase/NADH oxidase N-terminal domain-containing protein LOC123103353
*QDwt.uga-1D*	S1D_354847019	DWt_ GAP_24	C/T	0.378	8.14067 × 10^−5^	−0.04	**11.95**	**-**	
*QG.uga-2A*	S2A_43287532	G_GAP_25	T/A	0.465	4.73461 × 10^−5^	−0.45	7.84	TraesCS2A02G090400 TraesCS2A02G090500	Protein kinase domain-containing protein Protein SCAR, Protein WAVE LOC123184640
	S2A_43254369	G_GAP_25	G/T	0.471	6.84334 × 10^−5^	0.44	**14.45**	**-**	
*QGs.uga-2B*	S2B_638968494	GS_Across-environment BLUP	A/G	0.177	1.9189 × 10^−5^	−0.16	**10.05**	TraesCS2B02G446000	RRM domain-containing protein
		GS_GAP_24	G/A	0.177	7.26159 × 10^−6^	−0.36	**12.75**	TraesCS2B02G446100	Uncharacterized protein LOC123046478
								TraesCS2B02G446200	Uncharacterized protein LOC123046480
*QDwt.uga-3B*	S3B_666328969	DWt_Across-environment BLUP	G/C	0.041	7.9942 × 10^−6^	0.00	**10.61**	**-**	
*QG.uga-3B.2*	S3B_508983541	G_GAP_25	A/G	0.093	1.32666 × 10^−5^	0.95	**13.39**	**-**	
*QFht.uga-4A*	S4A_717822349	FHt_GAP_24	G/A	0.224	2.61163 × 10^−5^	−1.78	**12.66**	**-**	
*QGs.uga-6A*	S6A_606654266	GS_JPC_25	A/G	0.209	1.8762 × 10^−6^	0.00	**10.95**	**-**	
*QFwt.uga-6B.2*	S6B_572390420	FWt_GAP_24	G/A	0.169	2.77453 × 10^−5^	−0.29	**10.17**	TraesCS6B02G323400	CCT domain-containing protein, B-box-type zinc finger LOC123138142
*QFwt/Dwt.uga-6B.2*	S6B_574317560	FWt_GAP_24	C/T	0.166	1.4705 × 10^−5^	−0.30	**10.23**	**-**	
		DWt_GAP_24	T/C	0.166	5.4685 × 10^−5^	−0.05	9.15	-	
*QFht/Gs.uga-7A.2*	S7A_10270159	FHt_GAP_24	A/G	0.166	1.36395 × 10^−5^	−2.05	**12.03**	**-**	
		FHt_Across-environment BLUP	A/G	0.166	1.42987 × 10^−5^	−1.01	**11.65**	**-**	
		GS_Across-environment BLUP	G/A	0.166	9.959 × 10^−5^	−0.15	8.55	-	
*QFht.uga-7A*	S7A_19049097	FHt_GAP_24	G/C	0.166	9.59246 × 10^−5^	−1.93	**10.01**	**-**	
		FHt_Across-environment BLUP	C/G	0.166	4.76177 × 10^−5^	−0.99	**10.93**	**-**	
*QFht/Gs.uga-7A.3*	S7A_10867659	FHt_GAP_24	A/G	0.317	3.28539 × 10^−5^	−1.76	**14.47**	**-**	
	S7A_10909138	GS_Across-environment BLUP	C/T	0.113	1.3566 × 10^−5^	−0.19	9.43	TraesCS7A02G027200	Pentacotripeptide-repeat region of PRORP domain-containing protein LOC123148640
								TraesCS7A02G027300	USP domain-containing protein LOC123150293
*QGs.uga-7B*	S7B_887145	GS_GAP_25	A/C	0.035	2.17003 × 10^−5^	−0.37	**19.30**	TraesCS7B02G002300	Uncharacterized protein LOC123156299

^a^ QTL ID, Major-effect QTL identified in this study. ^b^ SNP associated with major-effect QTL. ^c^ Trait_Datasets include trait and environment combinations: G, germination counts; GS, Zadok’s growth stage; FHt, forage height (cm) at harvest; FWt, forage fresh weight in kg/ha; DWt, forage dry weight in kg/ha. GAP_24, BLUP values from Plains 2024 dataset; GAP_25, BLUP values from Plains 2025 dataset; JPC_25, BLUP values from J. Phil Campbell 2025 dataset; Across-environment BLUP, BLUP values from All-combined dataset. ^d^ Allele combination for the locus. ^e^ MAF, minor allele frequency. ^f^ *p* value. ^g^ Allelic effect on the trait. In GAPIT-based GWAS results, the effect is estimated for the second marker in alphabetical order, which means it could be a minor- or major-allele effect. ^h^ Percentage of phenotypic variance (PV) explained by the marker. Bold values indicate a PV explaining of ≥10%. ^i^ Candidate gene for QTL. ^j^ Protein produced by the candidate gene/Gene name.

**Table 4:** Allelic effect of significant markers associated with major-effect QTLs identified in this study.

QTL ^a^	SNP ^b^	Trait ^c^	MAF ^d^	Genotype ^e^	N ^f^	Mean BLUP ^g^	Tukey’s HSD Test ^h^
*QGs.uga-1A.4*	S1A_16789687	GS	0.122	CC	9	30.59	b
				CT	25	29.82	a
				**TT**	148	30.07	a
*QFwt.uga-1B.2*	S1B_526750430	FWt	0.113	**CC**	19	8368.48	b
				TC	1	9081.31	b
				TT	162	8065.77	a
*QFwt/Dwt.uga-1B.3*	S1B_534667041	FWt	0.116	CC	151	8056.00	a
				CT	2	8505.19	ab
				**TT**	19	8397.77	b
		DWt	0.116	CC	151	1425.67	a
				CT	2	1494.02	ab
				**TT**	19	1474.49	b
*QFwt.uga-1B.3*	S1B_533829236	FWt	0.105	CC	153	8065.77	a
				CT	2	8778.60	b
				**TT**	17	8388.01	b
*QDwt.uga-1D*	S1D_354847019	DWt	0.378	**CC**	63	986.25	b
				CT	7	1005.78	ab
				TT	112	908.13	a
*QG.uga-2A*	S2A_43254369	G	0.471	GG	78	85.87	a
				TG	6	85.19	a
				**TT**	88	86.78	b
*QFht.uga-4A*	S4A_717822349	FHt	0.224	**AA**	37	51.88	b
				GA	3	48.94	ab
				GG	132	48.24	a
*QGs.uga-7B*	S7B_887145	GS	0.035	AA	3	31.26	b
				CA	6	30.04	a
				**CC**	163	30.11	a

^a^ Major-effect QTL identified in this study. ^b^ SNP associated with major-effect QTL. ^c^ Forage yield and related traits. G, germination counts; GS, Zadok’s growth stage; FHt, forage height (cm) at harvest; FWt, forage fresh weight in kg/ha; DWt, forage dry weight in kg/ha. ^d^ MAF, minor allele frequency. ^e^ Homozygous and heterozygous genotypes for each SNP. The allele in bold is the favorable allele for the trait. ^f^ N, number of wheat lines having respective genotype class for each marker. ^g^ Mean BLUP value of traits in GAP_24, GAP_25, IHF_25, JPC_25, and All-combined. ^h^ Tukey’s HSD test results where different letters represent significant differences at *p* < 0.05.

## Data Availability

The original contributions presented in this study are included in the article and Supplementary Materials. Further inquiries can be directed to the corresponding author.

## Supplementary Materials

The following supporting information can be downloaded at https://www.mdpi.com/article/10.3390/cimb48100973/s1.
