# Supplementary material for: Dissecting the Genetics of Forage Yield and Related Traits in Soft Red Winter Wheat
Source: Curr Issues Mol Biol. 2026 Sep 22;48(10):973. doi: 10.3390/cimb48100973 (PMC13703133; doi:10.3390/cimb48100973)

Supplementary Table S1:

Descriptive statistics and analysis of variance (ANOVA) for the studied forage yield and related traits on 182 soft red winter wheat lines

Trait-Environment	General statistics of the population					ANOVA (p-value)				
	mean	SD	CV %	min	max	Genotype (G)	Replication (R)	GXE	EXR	Heritability
G_IHF_25	89.60	8.89	9.93	60	100	1	1	x	x	0
G_JPC_25	66.48	15.67	23.58	15	99	1	0.4171	x	x	0
G_GAP_25	86.33	7.82	9.06	60	100	0.11878	0.00139 **	x	x	0.2071818
G_GAP 24	x	x	x	x	x	x	x	x	x	x
G_All-combined	80.79	11.22	13.89	15	100	0.09681 .	x	1	< 2e-16 ***	0.09465142
GS_IHF_25	28.82	1.01	3.52	23	32	5.034e-05 ***	5.738e-10 ***	x	x	0.4551466
GS_JPC_25	25.27	0.97	3.84	23	28	0.9417	0.6396	x	x	0.01081628
GS_GAP_25	30.11	0.51	1.69	27	34	4.829e-10 ***	0.001257 **	x	x	0.6101241
GS_GAP_24	31.88	1.80	5.64	30	37	0.0001667 ***	6.146e-08 ***	x	x	0.43067337
GS_All-combined	29.02	1.17	4.03	23	37	3.405e-09 ***	x	0.0001683 ***	< 2e-16 ***	0.1720657
FHt_IHF_25	26.71	3.79	14.18	13	50	3.199e-08 ***	1	x	x	0.5649
FHt_JPC_25	21.19	2.11	9.96	16	27	1	0.1542	x	x	2.10e-09
FHt_GAP_25	36.43	4.95	13.57	18	62	6.225e-09 ***	6.897e-14 ***	x	x	0.5840855
FHt_GAP_24	48.88	5.77	11.81	23.6	68.1	1.636e-11 ***	4.903e-07 ***	x	x	0.6402298
FHt_All-combined	33.3	4.38	13.16	13.0	68.1	1.904e-14 ***	x	6.459e-14 ***	< 2e-16 ***	0.229965
FWt_IHF_25	4678.57	1975.74	42.23	556.59	13690.32	0.02963 *	1	x	x	0.2764165
FWt_JPC_25	8105.52	2312.09	28.52	3027.11	20525.72	0.1658	1	x	x	0.1860683
FWt_GAP_25	6886.21	2239.07	32.52	1201.08	12704.07	0.0006416 ***	0.1030362	x	x	0.3995997
FWt_GAP_24	5362.08	1969.03	36.72	839.78	12567.36	0.002163 **	3.752e-10 ***	x	x	0.36745488
FWt_All-combined	6258.1	2129.82	34.03	556.59	20525.72	0.0271309 *	x	0.0001392 ***	< 2e-16 ***	0.1816474
DWt _IHF_25	2724.21	761.68	27.96	1220.61	5312.08	1	0.03412 *	x	x	0
DWt_JPC_25	1434.87	367.20	25.59	527.30	3437.23	0.1806	1	x	x	0.1803782
DWt_GAP_25	2163.61	532.17	24.6	1132.72	3896.18	1	0.001468 **	x	x	0
DWt_GAP_24	935.74	323.98	34.62	175.77	1962.74	0.001066 **	1.769e-09 ***	x	x	0.3866726
DWt_All-combined	1814.61	529.86	29.2	175.77	5312.08	0.9604	x	0.5508	< 2e-16 ***	0.00212172

Supplementary Table S2:

Marker-trait associations (MTAs) identified for forage yield and related traits. These MTAs were resolved into 68 QTLs. The major-effect QTLs with phenotypic variance (PV) explained >10% are highlighted in bold.

QTL name	SNP	Trait	Environment	MAF	P.value	Effect	PV	Type
QGs.uga-1A.1	S1A_548956406	GS	GAP_25	0.148	1.21E-06	0.28	1.47	Minor
	S1A_549420673	GS	GAP_25	0.148	1.21182E-06	0.28	2.03	
	S1A_549287288	GS	GAP_25	0.151	1.59121E-06	0.27	2.02	
	S1A_549287294	GS	GAP_25	0.151	1.59121E-06	0.27	2.02	
	S1A_549295002	GS	GAP_25	0.154	1.69924E-06	0.27	1.50	
	S1A_548582464	GS	GAP_25	0.145	1.80065E-06	0.27	1.44	
	S1A_549422405	GS	GAP_25	0.154	3.05444E-06	0.26	1.50	
	S1A_549422427	GS	GAP_25	0.154	3.05444E-06	0.26	1.50	
	S1A_548995077	GS	GAP_25	0.157	3.67338E-06	0.25	1.50	
	S1A_549707037	GS	GAP_25	0.160	3.85224E-06	0.25	1.53	
	S1A_549298905	GS	GAP_25	0.151	4.49722E-06	0.25	1.47	
	S1A_549282971	GS	GAP_25	0.151	4.78049E-06	0.25	1.51	
	S1A_549314853	GS	GAP_25	0.108	5.99573E-06	0.28	4.64	
	S1A_549314870	GS	GAP_25	0.108	5.99573E-06	0.28	5.47	
	S1A_548929125	GS	GAP_25	0.148	6.12704E-06	0.26	2.54	
	S1A_548775419	GS	GAP_25	0.142	7.11086E-06	0.26	3.64	
	S1A_548683187	GS	GAP_25	0.145	1.00285E-05	0.26	5.50	
	S1A_549421316	GS	GAP_25	0.140	1.07892E-05	0.25	2.14	
	S1A_549421325	GS	GAP_25	0.140	1.07892E-05	0.25	2.14	
	S1A_549421345	GS	GAP_25	0.140	1.07892E-05	0.25	2.14	
	S1A_549421367	GS	GAP_25	0.140	1.07892E-05	0.25	2.14	
	S1A_549012098	GS	GAP_25	0.154	1.34661E-05	0.24	3.74	
	S1A_549422225	GS	GAP_25	0.148	1.62891E-05	0.24	3.01	
	S1A_549422245	GS	GAP_25	0.148	1.62891E-05	0.24	3.01	
	S1A_549422257	GS	GAP_25	0.148	1.62891E-05	0.24	3.01	
	S1A_549422263	GS	GAP_25	0.148	1.62891E-05	0.24	3.01	
QGs.uga-1A.2	S1A_557295935	GS	GAP_25	0.157	4.83878E-06	0.25	1.44	Minor
QGs.uga-1A.3	S1A_558316677	GS	GAP_25	0.148	1.21182E-06	0.28	1.47	Minor
	S1A_558253708	GS	GAP_25	0.154	3.89692E-06	0.25	1.54	
	S1A_558253736	GS	GAP_25	0.154	3.89692E-06	0.25	1.54	
	S1A_558071716	GS	GAP_25	0.151	4.49722E-06	0.25	2.02	
	S1A_558071774	GS	GAP_25	0.151	4.49722E-06	0.25	2.02	
	S1A_558487462	GS	GAP_25	0.151	4.78049E-06	0.25	1.51	
	S1A_558487471	GS	GAP_25	0.151	4.78049E-06	0.25	1.51	
	S1A_558070613	GS	GAP_25	0.154	4.86719E-06	0.25	2.08	
	S1A_558070622	GS	GAP_25	0.154	4.86719E-06	0.25	2.08	
	S1A_558483609	GS	GAP_25	0.157	8.80236E-06	0.23	1.50	
	S1A_558541135	GS	GAP_25	0.154	7.43572E-06	0.24	1.44	
	S1A_557977404	GS	GAP_25	0.154	1.53106E-05	0.24	1.96	
QGs.uga-1A.4	S1A_16789687	GS	GAP_25	0.122	5.21764E-06	-0.24	**12.82**	Major
QGs.uga-1A.5	S1A_560437999	GS	GAP_25	0.142	5.68043E-06	0.25	1.45	Minor
	S1A_559791086	GS	GAP_25	0.154	5.7599E-06	0.25	1.50	
	S1A_559976755	GS	GAP_25	0.157	6.04349E-06	0.24	1.54	
	S1A_560487941	GS	GAP_25	0.160	7.27164E-06	0.24	1.57	
	S1A_559780257	GS	GAP_25	0.154	1.21659E-05	0.24	1.96	
	S1A_559780854	GS	GAP_25	0.157	5.20278E-05	0.22	2.33	
	S1A_559886931	GS	GAP_25	0.166	4.77237E-05	0.21	1.78	
	S1A_559886932	GS	GAP_25	0.166	4.77237E-05	0.21	1.78	
QGs.uga-1A.6	S1A_547919073	GS	GAP_25	0.148	6.86049E-06	0.25	2.03	Minor
QGs.uga-1A.7	S1A_564314347	GS	GAP_25	0.154	3.05444E-06	0.26	1.50	Minor
	S1A_564314401	GS	GAP_25	0.154	3.05444E-06	0.26	1.50	
	S1A_564336284	GS	GAP_25	0.154	3.05444E-06	0.26	1.50	
QGs.uga-1A.8	S1A_566625886	GS	GAP_25	0.148	6.86049E-06	0.25	1.47	Minor
	S1A_566590619	GS	GAP_25	0.174	7.12798E-05	0.20	1.55	

QGs.uga-1A.9	S1A_551790671	GS	GAP_25	0.142	7.11086E-06	0.26	3.64	Minor
QGs.uga-1A.10	S1A_567520167	GS	GAP_25	0.154	3.05444E-06	0.26	1.50	Minor
	S1A_567520175	GS	GAP_25	0.154	3.05444E-06	0.26	1.50	
	S1A_568003154	GS	GAP_25	0.154	3.05444E-06	0.26	1.50	
	S1A_567384426	GS	GAP_25	0.160	6.02755E-06	0.24	1.53	
	S1A_568013275	GS	GAP_25	0.157	6.07517E-06	0.25	1.51	
	S1A_567478073	GS	GAP_25	0.151	1.63381E-05	0.24	2.44	
	S1A_568013220	GS	GAP_25	0.151	5.86935E-05	0.22	1.28	
QGs.uga-1A.11	S1A_570115379	GS	GAP_25	0.154	1.68296E-06	0.26	2.36	Minor
	S1A_570134061	GS	GAP_25	0.157	3.2694E-06	0.25	1.54	
	S1A_570134066	GS	GAP_25	0.157	3.2694E-06	0.25	1.54	
	S1A_570620103	GS	GAP_25	0.157	3.2694E-06	0.25	1.54	
	S1A_570620150	GS	GAP_25	0.157	3.2694E-06	0.25	2.13	
	S1A_570620203	GS	GAP_25	0.157	3.67338E-06	0.25	1.50	
	S1A_570620227	GS	GAP_25	0.157	3.67338E-06	0.25	1.50	
	S1A_570620233	GS	GAP_25	0.157	3.67338E-06	0.25	2.07	
	S1A_570423549	GS	GAP_25	0.157	3.97053E-06	0.25	1.54	
	S1A_570154544	GS	GAP_25	0.151	4.78049E-06	0.25	2.08	
	S1A_570392715	GS	GAP_25	0.151	1.14167E-05	0.25	2.21	
	S1A_570392750	GS	GAP_25	0.151	1.14167E-05	0.25	2.21	
	S1A_570615890	GS	GAP_25	0.160	1.51414E-05	0.23	2.73	
QGs.uga-1A.12	S1A_572072836	GS	GAP_25	0.154	2.21802E-06	0.26	1.92	Minor
	S1A_571959822	GS	GAP_25	0.154	3.05444E-06	0.26	1.50	
	S1A_572022482	GS	GAP_25	0.148	1.23612E-05	0.24	1.47	
QG.uga-1B.1	S1B_11408550	G	All-combined	0.282	1.43E-05	0.36	9.23	Minor
	S1B_12374440	G	All-combined	0.285	2.0239E-05	0.35	9.08	
	S1B_11881966	G	All-combined	0.276	2.4055E-05	0.35	8.65	
	S1B_12347619	G	All-combined	0.288	2.4059E-05	0.34	8.99	
QG.uga-1B.2	S1B_13895450	G	GAP_25	0.099	6.6528E-05	0.66	9.44	Minor
	S1B_14093773	G	All-combined	0.305	6.0119E-05	0.32	7.97	
	S1B_14093774	G	All-combined	0.305	6.0119E-05	0.32	7.97	
QFwt/Dwt.uga-1B.1	S1B_529627555	FWt	JPC_25	0.119	4.05352E-05	-0.56	9.12	Major
	S1B_529627861	FWt	JPC_25	0.119	1.22631E-05	-0.56	**10.56**	
		DWt	JPC_25	0.119	3.286E-05	-0.45	9.60	
	S1B_529854670	FWt	JPC_25	0.116	5.9459E-05	-0.55	8.80	
QG.uga-1B.3	S1B_17356959	G	All-combined	0.142	8.2217E-05	0.40	7.94	Minor
QFwt/Dwt.uga-1B.2	S1B_532396302	FWt	JPC_25	0.119	4.0535E-05	-0.46	**10.46**	Major
	S1B_531726815	FWt	JPC_25	0.113	3.2785E-05	-0.46	9.84	
	S1B_532401662	FWt	JPC_25	0.113	3.2785E-05	-0.46	**10.56**	
		DWt	JPC_25	0.116	6.5413E-05	-0.44	9.27	
		DWt	JPC_25	0.113	8.9694E-05	-0.48	8.76	
		DWt	JPC_25	0.113	8.9694E-05	-0.46	9.38	
	S1B_531867384	FWt	JPC_25	0.116	4.2252E-05	-0.55	**12.01**	
	S1B_532065919	FWt	JPC_25	0.116	4.2252E-05	-0.45	**12.01**	
	S1B_532400622	FWt	JPC_25	0.110	6.4413E-05	-0.54	**11.15**	
QG.uga-1B.4	S1B_12996948	G	All-combined	0.297	7.7343E-05	0.33	7.68	Minor
QFwt.uga-1B.1	S1B_531206150	FWt	JPC_25	0.116	4.54452E-05	-0.56	9.14	Minor
QGs.uga-1B	S1B_631718867	GS	GAP_25	0.497	6.75464E-05	-0.13	9.59	Major
	S1B_631718896	GS	GAP_25	0.497	6.75464E-05	-0.13	9.58	
	S1B_631816658	GS	GAP_25	0.485	9.40207E-05	-0.13	9.18	
	S1B_631816667	GS	GAP_25	0.485	9.40207E-05	-0.13	**11.06**	
QFwt.uga-1B.2	S1B_526750430	FWt	JPC_25	0.113	7.90363E-05	-0.45	**12.06**	Major
QFwt/Dwt.uga-1B.3	S1B_534667041	FWt	JPC_25	0.116	1.69826E-05	-0.67	**11.43**	Major
		DWt	JPC_25	0.116	4.5107E-05	-0.78	**10.37**	
QFwt.uga-1B.3	S1B_533829236	FWt	JPC_25	0.105	4.32498E-05	-0.82	**12.82**	Major
QG.uga-1B.5	S1B_29492494	G	All-combined	0.096	1.47E-05	0.62	**13.43**	Major
QG.uga-1B.6	S1B_9270181	G	All-combined	0.253	6.8426E-05	0.34	**10.64**	Major
QDwt.uga-1D	S1D_354847019	DWt	GAP_24	0.378	8.14067E-05	-0.04	**11.95**	Major
QG.uga-2A	S2A_43287532	G	GAP_25	0.465	4.73461E-05	-0.45	7.84	Major

	S2A_43254369	G	GAP_25	0.471	6.84334E-05	0.44	**14.45**	
QDwt.uga-2B	S2B_582802534	DWt	All-combined	0.203	7.1922E-05	0.00	8.25	Minor
	S2B_582802536	DWt	All-combined	0.203	7.1922E-05	0.00	8.25	
	S2B_582802537	DWt	All-combined	0.203	7.1922E-05	0.00	7.67	
	S2B_582802549	DWt	All-combined	0.203	7.1922E-05	0.00	8.25	
	S2B_582802557	DWt	All-combined	0.203	7.1922E-05	0.00	8.25	
QGs.uga-2B	S2B_638968494	GS	All-combined	0.177	1.9189E-05	-0.16	**10.05**	Major
		GS	GAP_24	0.177	7.26159E-06	-0.36	**12.75**	
QFwt.uga-2B.1	S2B_652825667	FWt	All-combined	0.267	7.6151E-05	-0.15	2.38	Minor
	S2B_652825691	FWt	All-combined	0.267	7.6151E-05	-0.15	2.38	
QFwt.uga-2B.2	S2B_652825716	FWt	All-combined	0.061	2.9283E-05	-0.16	6.71	Minor
QFwt.uga-2B.3	S2B_573669510	FWt	GAP_25	0.238	6.29132E-06	-0.83	2.65	Minor
QFwt.uga-2B.4	S2B_605381207	FWt	GAP_25	0.224	3.58614E-05	-0.91	0.77	Minor
QGs/Dwt.uga-2D	S2D_607986450	GS	GAP_24	0.087	5.60376E-05	0.57	2.32	Minor
	S2D_607986460	GS	GAP_24	0.087	5.60376E-05	0.57	2.32	
	S2D_607986475	GS	GAP_24	0.087	5.60376E-05	0.57	2.32	
	S2D_607986510	GS	GAP_24	0.087	5.60376E-05	0.57	2.32	
	S2D_607986522	GS	GAP_24	0.087	5.60376E-05	0.57	2.32	
		DWt	GAP_24	0.087	8.82686E-05	0.09	3.13	
		DWt	GAP_24	0.087	8.82686E-05	0.09	3.13	
		DWt	GAP_24	0.087	8.82686E-05	0.09	3.13	
		DWt	GAP_24	0.087	8.82686E-05	0.09	3.13	
		DWt	GAP_24	0.087	8.82686E-05	0.09	3.13	
QDwt.uga-3A	S3A_671739096	DWt	All-combined	0.416	3.2146E-05	0.00	5.83	Minor
QDwt.uga-3B	S3B_666328969	DWt	All-combined	0.041	7.9942E-06	0.00	**10.61**	Major
QG.uga-3B.1	S3B_790847303	G	All-combined	0.157	5.3096E-05	-0.55	1.24	Minor
QDwt/Fwt.uga-3B	S3B_655641665	DWt	JPC_25	0.427	2.3465E-06	-0.66	7.42	Minor
		FWt	JPC_25	0.427	1.0984E-05	-0.73	6.98	
QG.uga-3B.2	S3B_508983541	G	GAP_25	0.093	1.32666E-05	0.95	**13.39**	Major
	S3B_508983550	G	GAP_25	0.093	1.32666E-05	0.95	**13.39**	
	S3B_508983557	G	GAP_25	0.093	1.32666E-05	0.95	**13.39**	
QGs.uga-4A	S4A_694991707	GS	JPC_25	0.203	7.9888E-05	-0.00	2.39	Minor
	S4A_694991723	GS	JPC_25	0.203	7.9888E-05	-0.00	2.39	
QFht.uga-4A	S4A_717822349	FHt	GAP_24	0.224	2.61163E-05	-1.78	**12.66**	Major
	S4A_717822350	FHt	GAP_24	0.224	2.61163E-05	-1.78	**12.66**	
	S4A_717638968	FHt	GAP_24	0.148	7.5845E-05	-1.93	**10.67**	
QFht.uga-4B.1	S4B_241182089	FHt	JPC_25	0.017	5.7476E-05	0.00	6.81	Minor
QFht.uga-4B.2	S4B_241182111	FHt	JPC_25	0.017	5.7476E-05	0.00	9.07	Minor
QFwt.uga-5A.1	S5A_48764264	FWt	IHF_25	0.073	1.1851E-05	-0.29	7.15	Minor
	S5A_48968942	FWt	IHF_25	0.081	1.2455E-05	-0.29	4.04	
QFwt.uga-5A.2	S5A_47540564	FWt	IHF_25	0.064	1.5623E-05	-0.31	3.81	Minor
	S5A_47540601	FWt	IHF_25	0.064	1.5623E-05	-0.31	3.81	
QFwt.uga-5A.3	S5A_51560475	FWt	IHF_25	0.084	4.1701E-05	-0.27	6.54	Minor
QFwt.uga-5A.4	S5A_49695748	FWt	IHF_25	0.078	4.7816E-05	-0.27	6.57	Minor
QFwt.uga-5A.5	S5A_46366358	FWt	IHF_25	0.076	6.5647E-05	-0.27	6.17	Minor
QFwt.uga-5B.1	S5B_665951143	FWt	All-combined	0.206	1.2932E-05	0.13	3.08	Minor
QFwt.uga-5B.2	S5B_670020353	FWt	All-combined	0.291	2.1083E-05	0.10	4.81	Minor
	S5B_670020354	FWt	All-combined	0.291	2.1083E-05	0.10	4.47	
	S5B_670020362	FWt	All-combined	0.291	2.1083E-05	0.10	4.47	
	S5B_670020384	FWt	All-combined	0.291	2.1083E-05	0.10	4.47	
	S5B_670020387	FWt	All-combined	0.291	2.1083E-05	0.10	4.81	
	S5B_670020412	FWt	All-combined	0.291	2.1083E-05	0.10	4.47	
QFwt.uga-5B.3	S5B_669020878	FWt	All-combined	0.276	4.4413E-05	0.10	3.80	Minor
	S5B_669020880	FWt	All-combined	0.276	4.4413E-05	0.10	3.80	
QGs.uga-6A	S6A_606429707	GS	JPC_25	0.206	1.2921E-05	0.00	8.43	Major
	S6A_606654266	GS	JPC_25	0.209	1.8762E-06	0.00	**10.95**	
QFwt/Dwt.uga-6B.1	S6B_568869924	FWt	GAP_24	0.160	3.21618E-05	-0.30	8.87	Minor
	S6B_568869926	FWt	GAP_24	0.160	3.21618E-05	-0.30	8.87	
	S6B_568869929	FWt	GAP_24	0.160	3.21618E-05	-0.30	8.87	

	S6B_568869930	FWt	GAP_24	0.160	3.21618E-05	-0.30	8.87	
	S6B_568869933	FWt	GAP_24	0.160	3.21618E-05	-0.30	8.87	
		DWt	GAP_24	0.160	7.79795E-05	-0.05	8.69	
		DWt	GAP_24	0.160	7.79795E-05	-0.05	8.69	
		DWt	GAP_24	0.160	7.79795E-05	-0.05	8.69	
		DWt	GAP_24	0.160	7.79795E-05	-0.05	8.69	
		DWt	GAP_24	0.160	7.79795E-05	-0.05	8.69	
QFwt.uga-6B.1	S6B_571407714	FWt	GAP_24	0.157	7.50774E-05	-0.29	7.91	Minor
	S6B_571407745	FWt	GAP_24	0.157	7.50774E-05	-0.29	7.91	
QFwt.uga-6B.2	S6B_572390420	FWt	GAP_24	0.169	2.77453E-05	-0.29	**10.17**	Major
	S6B_572390390	FWt	GAP_24	0.163	7.32451E-05	-0.28	9.28	
QFwt/Dwt.uga-6B.2	S6B_574317560	FWt	GAP_24	0.166	1.4705E-05	-0.30	**10.23**	Major
		DWt	GAP_24	0.166	5.4685E-05	-0.05	9.15	
	S6B_574317582	FWt	GAP_24	0.160	3.97716E-05	-0.29	9.27	
QFwt.uga-6B.3	S6B_570698746	FWt	GAP_24	0.166	2.4287E-05	-0.30	9.76	Minor
QFwt.uga-6B.4	S6B_569389830	FWt	GAP_24	0.166	4.36928E-05	-0.29	8.55	Minor
QDwt.uga-6B	S6B_570698746	DWt	GAP_24	0.166	8.97218E-05	-0.05	8.40	Minor
QGs/Fht.uga-7A.1	S7A_38234502	GS	GAP_24	0.052	4.96531E-05	-0.48	6.07	Minor
	S7A_38242937	GS	GAP_24	0.052	4.96531E-05	-0.48	6.07	
	S7A_38327857	GS	GAP_24	0.052	4.96531E-05	-0.48	6.07	
		FHt	All-combined	0.052	4.156E-05	-1.34	8.24	
		FHt	All-combined	0.052	4.156E-05	-1.34	8.24	
		FHt	All-combined	0.052	4.156E-05	-1.34	8.24	
		GS	All-combined	0.052	6.7093E-05	-0.22	6.38	
		GS	All-combined	0.052	6.7093E-05	-0.22	6.38	
		GS	All-combined	0.052	6.7093E-05	-0.22	6.38	
QFht/Gs.uga-7A.2	S7A_10270159	FHt	GAP_24	0.166	1.36395E-05	-2.05	**12.03**	Major
	S7A_10270169	FHt	GAP_24	0.166	1.36395E-05	-2.05	**12.03**	
		GS	All-combined	0.166	9.959E-05	-0.15	8.55	
		GS	All-combined	0.166	9.959E-05	-0.15	8.55	
		FHt	All-combined	0.166	1.42987E-05	-1.01	**11.65**	
		FHt	All-combined	0.166	1.42987E-05	-1.01	**11.65**	
QGs.uga-7A.1	S7A_128784756	GS	GAP_24	0.471	8.42361E-05	0.24	3.85	Minor
QFht.uga-7A	S7A_19049097	FHt	GAP_24	0.166	9.59246E-05	-1.93	**10.01**	Major
		FHt	All-combined	0.166	4.76177E-05	-0.99	**10.93**	
QFht/Gs.uga-7A.3	S7A_10867659	FHt	GAP_24	0.317	3.28539E-05	-1.76	**14.47**	Major
	S7A_10909138	GS	All-combined	0.113	1.3566E-05	-0.19	9.43	
	S7A_10909175	GS	All-combined	0.113	1.3566E-05	-0.19	9.43	
QGs.uga-7A.2	S7A_127910397	GS	GAP_24	0.485	7.79247E-05	0.24	3.94	Minor
	S7A_128056303	GS	GAP_24	0.468	9.57425E-05	0.24	4.65	
QGs.uga-7A.3	S7A_608939900	GS	All-combined	0.372	5.24166E-05	0.12	2.55	Minor
	S7A_608939899	GS	All-combined	0.369	8.13836E-05	0.12	2.65	
QGs.uga-7B	S7B_887145	GS	GAP_25	0.035	2.17003E-05	-0.37	**19.30**	Major
QFht.uga-7B	S7B_1384484	FHt	GAP_25	0.186	1.5269E-05	-1.53	7.92	Minor
	S7B_1384488	FHt	GAP_25	0.186	1.5269E-05	-1.53	7.92	
	S7B_1384503	FHt	GAP_25	0.186	1.5269E-05	-1.53	7.92	
	S7B_1384504	FHt	GAP_25	0.186	1.5269E-05	-1.53	7.92	
	S7B_1384510	FHt	GAP_25	0.186	1.5269E-05	-1.53	7.92	
	S7B_1384513	FHt	GAP_25	0.186	1.5269E-05	-1.53	7.76	

Supplementary Figure S1: Correlation analysis of GS and forage yield and related traits among 182 SRWW lines, single four environment datasets a) GAP_24: b) GAP_25: c) IHF_25: d) JPC_25. G, germination percentage; GS, Zadok's growth stages; FHt, forage height ta harvest in cm; FWt, forage fresh weight in kg/ha; DWt, forage dry weight in kg/ha; *** significant at $p<0.001$, ** significant at $p<0.01$, * significant at $p<0.05$ probability level.

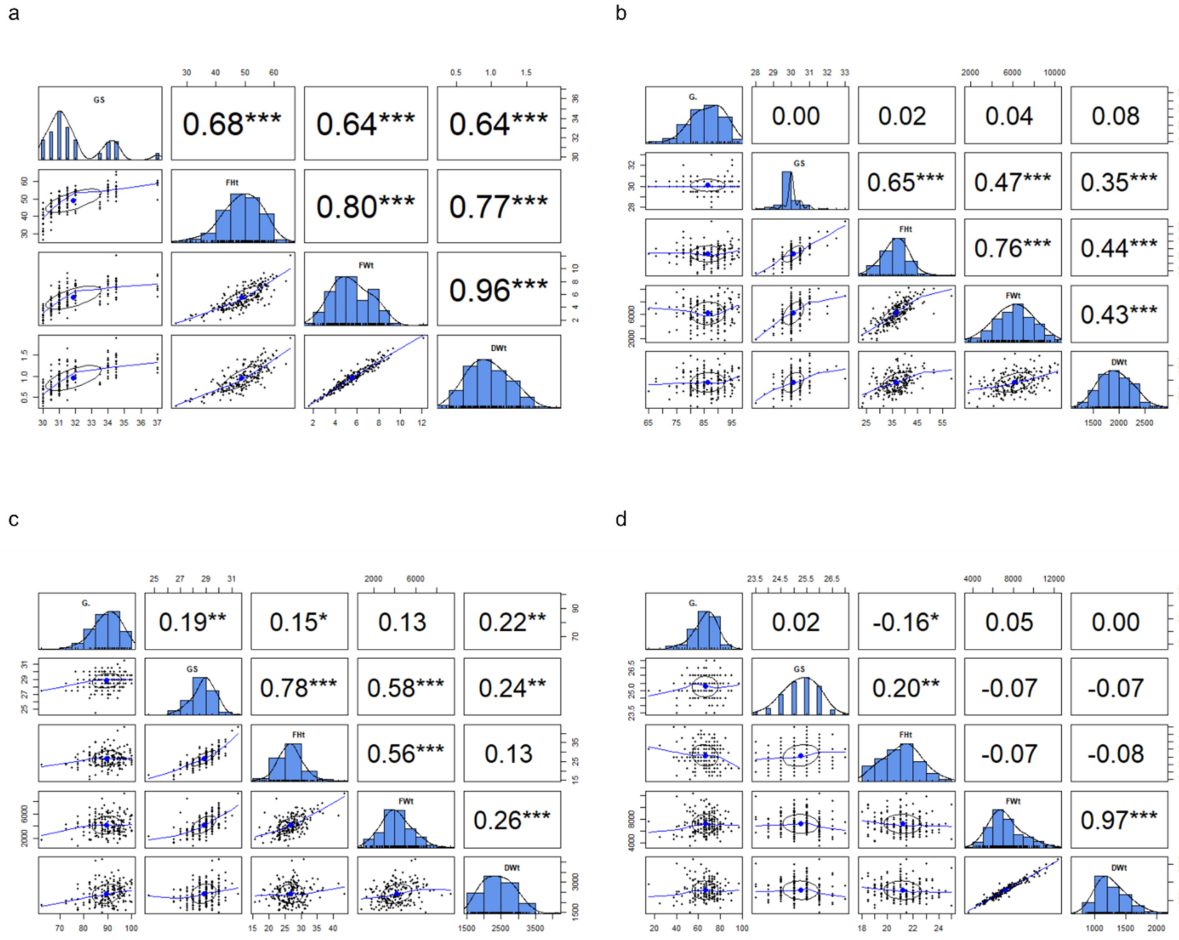

Supplementary Figure S2: Scatter plot showing linkage disequilibrium (LD) across the wheat genome and each chromosome. The r^2 of pair of single nucleotide polymorphisms (SNP) was plotted against the physical distance in base pairs (bp). The numbers in the blue show the LD decay distance at the LD population threshold of 0.2.

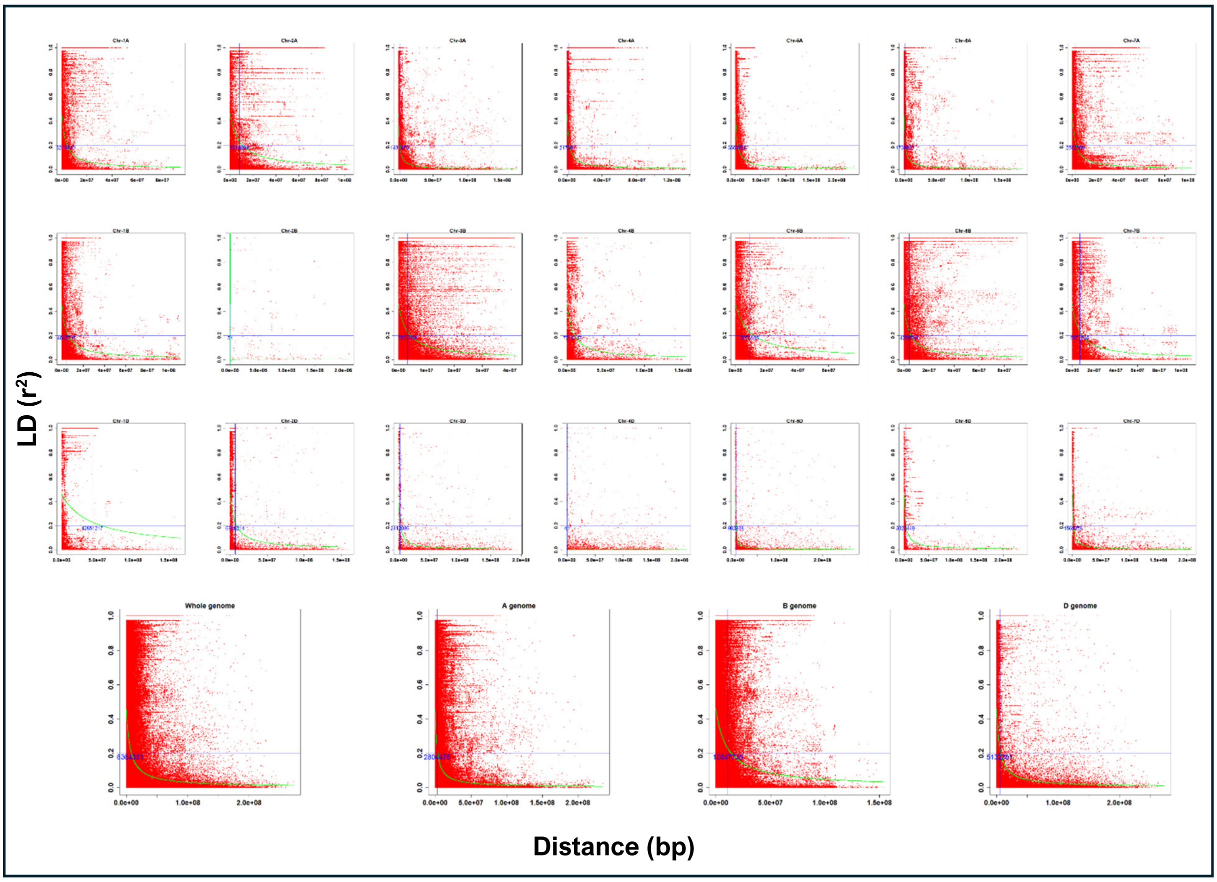

Supplementary Figure S3: Comparison of four different GWAS models for forage fresh weight (FWt) (a) and forage dry weight (DWt) (b) from JPC_25 environment. GLM, generalized linear model; MLM, mixed linear model; MLMM, multiple loci mixed model; FarmCPU, fixed and random model circulating probability unification.

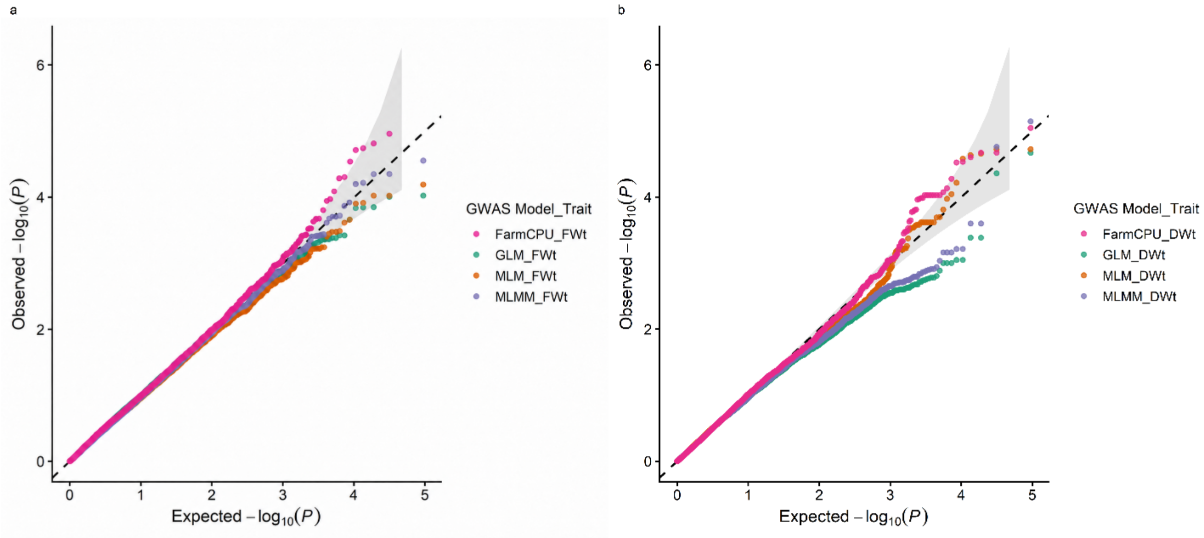

Supplement: Supplementary file 1 [file cimb-48-00973-s001.zip › cimb-4527735-supplementary.pdf]
